# Female-friendly toilets in schools in Burkina Faso: A mixed-methods study using photo-elicitation

**DOI:** 10.7189/jogh.12.04057

**Published:** 2022-09-08

**Authors:** Teresa Buitrago-García, N Hélène Sawadogo, Aurélia Souares, Jean-Louis Koulidiati, Ali Sié, Till Bärnighausen, Sarah Langlotz, Shannon A McMahon

**Affiliations:** 1 Heidelberg Institute of Global Health, Heidelberg University Hospital, Heidelberg, Germany; 2 La Paz University Hospital, Madrid, Spain; 3 Nouna Health Research Centre, Nouna, Burkina Faso; 4 Africa Health Research Institute, Nelson R. Mandela Medical School, Umbilo, Durban, South Africa; 5 Department of Global Health and Population, Harvard T.H. Chan School of Public Health, Boston, Massachusetts, USA; 6 Chair of Development Economics (Prof. Fuchs), Georg-August-Universität Göttingen, Göttingen, Germany; 7 Bloomberg School of Public Health, Johns Hopkins University, Baltimore, Maryland, USA

## Abstract

**Background:**

An absence of gender-sensitive sanitation facilities in schools and the negative effects this has on girls has been widely discussed among advocacy groups, though less examined in academic spheres. Drawing on triangulated data, we outline current challenges and respondent-driven solutions to enhance the female-friendly nature of toilets in a context of extreme poverty.

**Methods:**

This mixed-methods study was informed by the tenets of human-centred design. We first quantitatively assessed facilities in 14 secondary schools in the Kossi Province of Burkina Faso. We then collected qualitative data, including 15 focus group discussions and 53 in-depth interviews among schoolgirls, mothers, teachers and key informants. We applied photo-elicitation, a novel method, to explore perceptions of facilities and the desirability and feasibility of interventions to improve gender-friendly sanitation facilities.

**Results:**

No school met international water, sanitation and hygiene (WASH) standards for schools. Roughly one third of schools did not have water and, when present, there was no reliable way to use it within the toilet complex. Schoolgirls shared feelings of shame and stress when menstruating at school, and said that they would avoid using school toilets, if possible. Schoolgirls described water access as the most urgent need to address, followed by fostering privacy and facilitating cleanliness within facilities. Mothers and teachers mostly aligned with these priorities, while key informants additionally emphasised the need to raise awareness on both general and menstrual hygiene and to develop maintenance systems. Photo-elicitation engaged and empowered participants to pinpoint priorities and concrete solutions, namely a need for doors and locks, water containers and cleaning materials.

**Conclusions:**

WASH needs in many schools remain unmet. Women and girls should be involved in decision-making across stages of intervention design and implementation. Young women’s voices merit greater inclusion in academic literature. Future interventions should enhance access to water and privacy. Future research could explore maintenance and monitoring strategies to develop guidance on sustainable solutions.

Female-friendly toilets have been described as gender-segregated facilities that provide safety, privacy, lighting, water, soap and a culturally appropriate way to dispose of menstrual waste. Such toilets must have features desired in all toilets (eg, suitable drainage systems), while also being attuned to gendered needs (eg, being safely accessible by day and night, and allowing for safe disposal of menstrual products) [1]. Recent calls for an expansion of female-friendly toilets stem from a recognition that women and girls are disproportionately affected by poor sanitation [2]. An absence of female-friendly toilets contributes to negative health outcomes among women and girls including: an increased risk of violence [3], psychosocial stress [4,5], urogenital and reproductive tract infections [6,7] and other social and health issues that arise from inadequate water, sanitation and hygiene (WASH) [8].

In the school context, a lack of WASH facilities is regarded as a contributor to higher rates of repetition and dropout among girls [9]. Girls themselves describe how inadequate WASH options spark anxiety because of concerns about stains on clothing, pain and discomfort associated with toilet avoidance and fears about peer harassment [10]. Interventions that entail construction or expansion of female-friendly facilities in schools have been shown to increase girls’ enrolment [10], attendance [11] and completion rates [12].

Nearly 900 million children worldwide lack basic hygiene services at their school [13]. Existing WASH standards for schools [14], developed over a decade ago, include guidance regarding water access, hygiene, cleaning and waste disposal, control of vector-borne diseases and food storage. For bathrooms in particular, WASH standards recommend a 25:1 pupil-to-toilet ratio for girls (50:1 plus one urinal for boys) along with privacy and safety, gender segregation, handwashing facilities, daily cleaning and disinfection, and accessibility for people with disabilities. The standards include no guidance regarding menstrual needs. Guidelines for female-friendly public toilets have been developed more recently [15], but are not fully applicable to school toilets because communal, public toilets vary in terms of users, location, opening hours, etc.; these factors limit the ability to extrapolate recommendations. We are not aware of peer-reviewed literature outlining how schoolgirls and their teachers describe existing or ideal toilet conditions in low- and middle-income countries (LMICs), although gathering insights from end-users is essential to improve products and services [16].

This study fills a gap in the literature by applying a novel research method (photo-elicitation) to examine an understudied topic (female-friendly toilets) in an understudied LMIC setting (semi-urban and rural Burkina Faso). Along with capturing data regarding the state of female friendly toilets in this setting, we also capture schoolgirls' opinions of female-friendly toilets, and schoolgirls' preferences in terms of enhancing the acceptability and usability of toilets.

## METHODS

### Setting

Burkina Faso, a landlocked country in Sub-Saharan Africa, is among the lowest 5% of countries in terms of a Human Development Index [17]. With regards to gender equity, Burkina Faso ranks 129 of 153 with inadequate opportunities for women in sectors related to health, education, economic status and political representation [18]. According to a country-representative survey in 2018, an estimated 34.9% of women aged 15-24 have never attended secondary school, and more than half of girls in rural areas are married by age 18 [19]. Only a quarter of Burkinabe women nationally report having the resources they need to manage menstruation [20]. At a national level, 44% of secondary schools have no water services and only 53% have improved, usable, single-sex bathrooms [21]. Our study site is in the Health and Demographic Surveillance System (HDSS) located in the semi-urban area of Nouna and the 58 surrounding villages, in the northwest region of Boucle du Mouhoun, 290 km from the capital Ouagadougou. In the region, an estimated 27% of towns have hand washing facilities and 58% of people report using latrines [22]. We chose this setting because more research is needed in francophone West Africa [23], in contexts of extreme poverty, and particularly in more rural areas [24]. Furthermore, the Nouna Health Research Centre and the Heidelberg Institute of Global Health have engaged in a longstanding collaboration for decades, which facilitated mutual trust and scientific rigor.

### Design and sampling

Our work followed the principles of human-centred design, a holistic approach that focuses on human needs and seeks to find usable and useful solutions that fit the complex dynamics of users’ reality, based on context, co-creation and iteration [25]. This framework is growingly adopted in global health [26], and has been previously used in research related to handwashing [27,28] and transformative WASH [29]. For more information on our methods, see Appendix S1 in the **Online Supplementary Document**.

**Figure 1:**
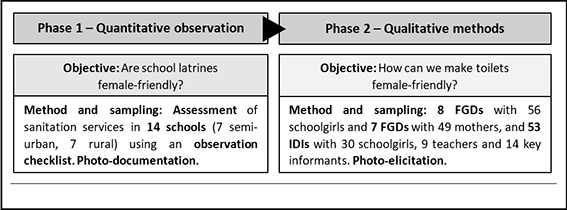
Study design overview.

This study included two main phases (**Figure 1**), beginning with structured, quantitative observations, followed by personal engagement via focus group discussions (FGDs) and in-depth interviews (IDIs). Schools and surrounding settings were chosen based on their inclusion in the Nouna HDSS. All sampling was purposive [30].

### Data collector training

Six local researchers participated in a 5-day training, which covered topics including: WASH, female health related to sanitation (biological, sociocultural and environmental issues), qualitative and quantitative methods, data debriefings and research ethics. Local researchers had all previously attended research trainings at the Nouna Health Research Centre and several researchers had backgrounds in the social sciences, ranging from political science to pedagogy. Investigators became acquainted with research tools, practised debriefings, piloted interview guides and refined guides before embarking on data collection [31]. A written manual was created for data collectors to use as a reference during data collection (see Appendix S2 in the **Online Supplementary Document**).

### Data collection

In a first phase of research in 2018, we conducted structured observations and photo-documentation of WASH facilities in all secondary schools (n = 14) within a 40 km radius of Nouna town. We evaluated facilities using an observation sheet based on the tools in “WASH in Schools Empowers Girls’ Education” [32], which covers water, sanitation, hygiene and waste disposal with regards to menstrual needs (see Appendix S3 in the **Online Supplementary Document**). If separate toilets for teachers were present, we assessed them in the same manner. School visits lasted 40 minutes on average.

In a second phase of research in 2019, we used typical case sampling [30] to identify two schools that were representative of the other schools in that they had scored “average” in relation to WASH facilities (based on structured observations in phase 1). Within these two schools (one private, one public), we conducted FGDs with schoolgirls and their mothers.

Concurrently, we conducted IDIs with key informants (KIs) including school principals, religious leaders, national health and education officers and representatives from non-governmental organisations engaged in menstruation-related activities. We further sampled teachers considering gender, school location and duration of work experience in each institution. **Table 1** describes the demographic characteristics of participants. **Table 2** describes inclusion criteria of all participants.

**Table 1:** Demographic characteristics of participants

	Schoolgirls	Mothers	Teachers	KI
**Participant sample**	n = 56 / n = 30	n = 49	n = 9	n = 14
**Research activities**	8 FGDs / 30 IDIs*	7 FGDs	9 IDIs	14 IDIs
**Age (years)**				
Mean	17.41 (n = 56)	39.22 (n = 46)	32.17 (n = 6)	-
Median	17	37	31.5	-
Range	12-28	19-67	28-39	-
**Gender (n)**				
Female	56	49	6	6
Male	0	0	3	8
**Religion (n)**				
Muslim	32	29	2	-
Catholic	18	17	4	-
Christian	6	2	0	-
Animist	0	1	0	-
Unknown	0	0	3	-
**Marital status (n)**				
Single	54	4	2	-
Married	2	41	4	-
Widowed	0	3	0	-
Divorced	0	1	0	-
Unknown	0	0	3	-
**Level of education (n)**				
None	-	29	-	-
Attended primary	-	4	-	
Completed primary	-	7	-	-
Attended secondary	-	2	-	-
Completed secondary	-	7	-	-
**Schooled daughters in household (n)**				
Mean	-	2.29	-	-
Median	-	2	-	-
Range	-	1-6	-	-
**Work experience (years)**				
Mean	-	-	7.17 (n = 6)	12.08 (n = 12)
Median	-	-	8	11
Range	-	-	4-9	1-36
**Field of expertise – n**				
WASH in Burkina Faso	-	-	-	3
MHH in Burkina Faso	-	-	-	3
MHH in schools	-	-	-	3
Sociocultural beliefs	-	-	-	3
Healthcare	-	-	-	2

*56 schoolgirls participating in 8 FGDs, with 30 of them taking part in 30 IDIs.

WASH – water, sanitation and hygiene, MHH – menstrual health and hygiene, KI – key informants, FGD – focus group discussions, IDI – indepth interviews

**Table 2:** Research activities and inclusion criteria

Respondent group	Inclusion criteria
**Schoolgirls FGD**	≥12 y old, with parental and schoolgirl’s informed consent, post-menarchal
**Mothers FGD**	≥18 y old, providing informed consent, with an underage daughter participating in preceding FGDs
**Schoolgirls IDI**	≥12 y old, with parental and schoolgirl´s informed consent, participation in preceding FGDs, willing to try a new sanitary product and available for follow-up
**Semi-urban and rural school teachers IDI**	≥18 y old, providing informed consent, slightly oversampling female perspectives, working in schools in our sample, with longest permanence in each institution
**Local key informants IDI National key informants IDI**	≥18 y old, providing informed consent, with leading role in education, health and socio-religious domains in national institutions and non-governmental organisations, with experience in MHM

FGD – focus group discussions, IDI – in-depth interviews

Although qualitative guides contained a similar amount of questions, FGDs with mothers took considerably longer than with girls (median 155 minutes vs 100 minutes, respectively). Furthermore, IDIs with teachers generally lasted longer (around 90 minutes) compared to IDIs with schoolgirls and KIs (approximately 60 minutes).

### Photo-elicitation

**Figure 2:**
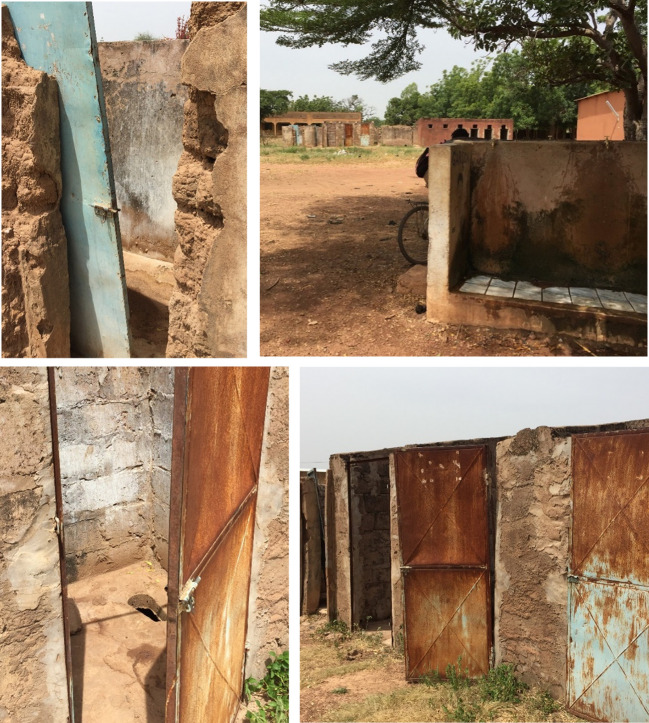
Set of pictures A, as used in photo-elicitation: Images show a shower space without water, the school’s water source, a latrine and the students’ toilet block.

**Figure 3:**
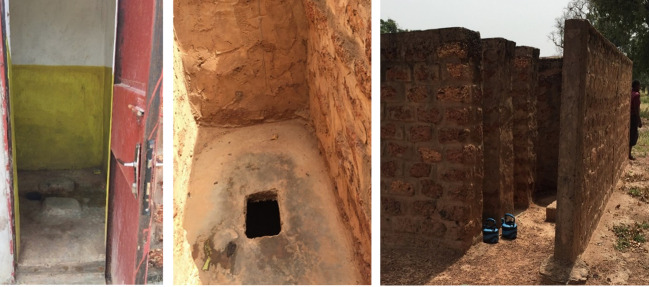
Set of pictures B, as used in photo elicitation: Images show a teachers’ toilet, a students’ toilet and the students’ toilet block (kettles shown beside them).

Photo-elicitation, which was incorporated into the qualitative component of this research, is a relatively underused technique to draw out insights by discussing photographs. The approach is grounded in an understanding that pictures can evoke a different and more nuanced dialogue than questions alone [33]. The method has been shown to facilitate verbalisation and insight, and to encourage younger participants to take the lead and express themselves [34]. While we are not aware of photo-elicitation being used in studies of school toilet facilities, the technique has enriched and facilitated interview processes in research throughout sub-Saharan Africa on topics including resilience [35], gender [36,37], pain [38] and palliative care [39,40]. We used photo-elicitation to generate a detailed understanding of girls and other stakeholders’ perceptions of toilets. We selected pictures for photo-elicitation from images gathered during the first, observational phase of data collection, excluding pictures from the two schools where the second phase of research took place. We presented images that reflected a “typical case” school, based on the features observed during school visits and the scores obtained (see “Appendices S3 and S4” in the **Online Supplementary Document**). We excluded images that depicted exceptionally high (eg, flush toilets) or low (eg, toilets without walls) quality toilets, that were seen in some schools but did not represent the norm. Photos were shown in two sets (see **Figure 2** and **Figure 3**). While each set was shown, respondents were asked to describe the pictures, identifying what they liked or disliked, and to compare the toilets to their own at school or at home.

### Data analysis

Analysis began in the field via daily debriefings, where the data collection team discussed arising themes and areas of improvement [31]. All interviews were digitally recorded, verbatim transcribed and translated into French, when applicable. All transcripts were verified by bilingual research assistants and coded using thematic analysis by two members of the research team. We applied triangulation for coding and compared across information sources (observations, FGDs and IDIs) and respondent groups (schoolgirls, mothers, teachers and KIs) to gauge how respondent priorities aligned with or refute WASH standards. The coding process was supported by Nvivo 12 software (QSR international, Burlington MA, USA).

### Ethics

The study received approval by the local ethics committee at the research centre in Nouna (2018-015-/CIE/CRSN) and the ethics committee of the Medical Faculty of Heidelberg University in Germany (S-654/2018). At the time of the study, the national ethical committee was not functioning in Burkina Faso. We obtained informed, written consent from all participants, as well as their parents, in the case of underage schoolgirls.

## RESULTS

### Phase 1. School Observations

Of the 14 schools included in this study, none met the minimum WASH standards for schools in low-income settings [14]. Girls represented 46.16% (3180) of the total student population (6892); approximately 20% of schools did not have any female teachers. For a breakdown of demographic details across schools and an assessment of their sanitation facilities, see Appendix S4 in the **Online Supplementary Document**.

In terms of water and sanitation, one third of schools (n = 5) lacked any water source. When present, water sources were usually of a faucet-type, although there were also schools with manual pumps and wells. Water was in all cases located too far to be used within toilet stalls, and would have required a bucket or container (eg, a plastic kettle) to carry it. We observed kettles readily available for toilet use in one school. None of the schools had functioning handwashing facilities nor did schools provide soap, sanitary products in case of emergency, or pain medications (eg, ibuprofen). One school had cement water containers for handwashing in front of toilets that had been installed during a prior intervention, but they were out of order at the time of the visit. Two schools had water pumps that were not functioning. No school had designated places for disposal of sanitary products nor trash cans located near or inside toilets. Trash was typically left on the floor of toilets, or on school grounds. In terms of toilets, the median pupil to toilet ratios was 68.9:1 (range 34.3 to 352.3:1). No school met the international standard ratio of 25:1 [14]. All students and teachers’ toilets were pit latrines, except for flush toilets for teachers in two schools. More than half of all schools (n = 9) did not have had sex-divided bathrooms. Regarding cleanliness, many schools (n = 8) had toilets with urine, excrement or other kinds of dirt outside of the pits. Half of the schools (n = 7) had toilet stalls with doors and mostly functional locks, but they were often rusty and difficult to close. Interiors of all bathrooms were bare, with no mirrors to check for blood stains and no hooks or shelves where girls could place their sanitary products or hang clothes. No school had bathrooms adapted for students with disabilities.

Nearly all schools (n = 10) provided separate toilets with functioning doors and locks (n = 9) for teachers and administrative staff, but few were sex-divided (n = 3), contrary to World Health Organisation recommendations [14]. The average ratio of teachers per toilet was 14.6:1 (ranging from 2.5 to 61:1) and cleanliness scores were higher. No teacher facilities were adapted for staff with disabilities.

### Phase 2. Qualitative data, including photo-elicitation

Upon being asked to comment on the photos or to share experiences and perceptions more generally, schoolgirls’ emphasized the personal challenges they face related to water, cleanliness and privacy. Mothers and teachers echoed these concerns and empathized with schoolgirls, with teachers expressing either resignation or unease when commenting on bathrooms in their own schools. One female teacher refused to comment altogether saying that she was new to the school, and she had never gone into them. All adults interviewed noted that students would not likely feel comfortable using toilets such as those in the photos because they were “too dirty” (Mother, age 34), “do not preserve their privacy” (Male teacher, age 28) and “do meet any hygienic standards” (Male health district informant, age 36). Some mothers, however, noted that toilets in some of the pictures were overall better than the toilets used at home, although not regarding cleanliness. Multiple KIs described toilets portrayed in photos with similar words, such as “unsafe”, “not adapted”, “not well maintained”, offering “no privacy”, and therefore, “not suitable for managing periods at school”. However, KIs focused more on challenges regarding financial scarcity and personal experiences implementing interventions to address hygiene.

**Figure 4:**
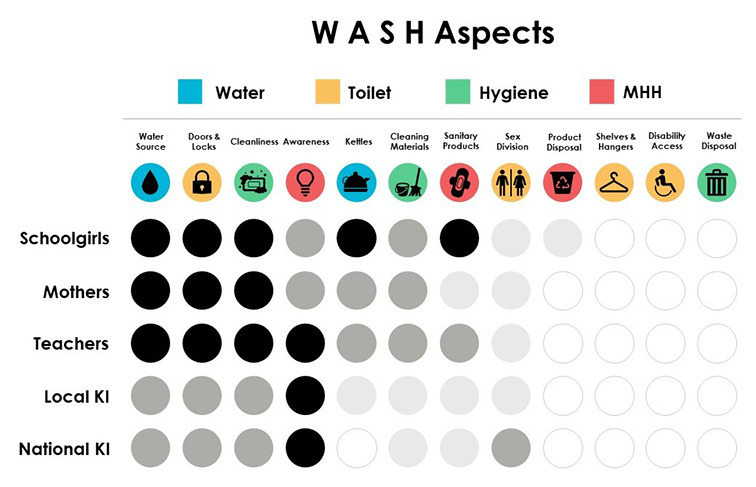
WASH framework priorities as discussed by participants. Icon credits: Courtesy of www.flaticons.com: WASH – water, sanitation and hygiene, MHH – menstrual health and hygiene, KI – key informants. Black circle – discussed by almost all, Dark grey circle – discussed by some, Light grey circle – mentioned, White circle – mentioned by none/almost none.

**Figure 4** presents an overview of WASH topics as discussed across respondent groups, highlighting that no single topic dominated all interactions. Some issues sparked more intense dialogue (eg, debates related to water accessibility, doors and locks, cleanliness and menstrual hygiene awareness). In some cases, national level stakeholders described issues that went unmentioned by all others (namely, the inclusion of shelves and hangers in bathrooms). Disability access was mentioned by one national KI. In other cases, issues that were of exceptional importance to girls, went unmentioned by national-level informants (eg, having water kettles). Schoolgirls described with conviction their need for sanitary products, but this was less emphasized in conversations with mothers, teachers and most KIs. Sanitary product disposal was mentioned occasionally, but not brought up as a priority recommendation to improve toilets as it was socially accepted to throw pads into the pits, as long as they were deep enough. No respondent group spoke about challenges or experiences with waste disposal.

### Theme 1. Cleanliness and access to water

Schoolgirls described toilets in pictures as “dirty”, “disgusting”, “smelly” and “broken”. When asked whether they would use the toilets in pictures, all girls agreed that this would cause discomfort with one girl saying, “You already feel disgusting on your period and you come into a toilet that is again dirty, you feel even more disgusted” (Schoolgirl, age 15). Mothers were also most concerned about cleanliness and the provision of cleaning materials, but conversations with them focused more on other topics outside of WASH, such as fear of unwanted pregnancies and pain management during menstruation.

The lack of cleanliness drove some teachers to discourage toilet use as they perceived it a risk for infections: “I like to tell girls that a girl cannot randomly go into a toilet. If you can hold it, you should wait until you get home. Women’s genitalia take everything. You have to sit down to urinate and the dirt goes up and enters your vagina” (Female teacher, age 31).

Girls described the essential need for water as a means to keep themselves and their toilets clean, and while they noted that water existed on school grounds in many schools (including their own) it was effectively inaccessible because there was no way to transport water to a toilet from the main well or fountain in the school. Girls used words such as “endure”, “stressful” and “suffer”, when describing how they tried to go about the school day while needing to use the toilet. Conversations about water and cleanliness also intertwined with girls’ conversations about menstruation; several schoolgirls described the impossibility of effectively cleaning themselves and maintaining privacy when menstruating at school. “If I'm on my period at school, I'm not safe to change, there is no kettle to put water in (…) and it is often dirty” (Schoolgirl, age 16). Schoolgirls consistently remarked on the value of plastic kettles (shown in **Figure 3**), and expressed their appreciation that kettles were placed directly next to the toilets in the photos. Teachers described how the norm in many schools is for kettles to be kept by teachers or administrative staff, because “if we leave them outside, we risk losing them” (Female teacher, age 31). The act of asking for a kettle sparks embarrassment because it is a sign that one either needs to defecate or is menstruating – both taboo topics.

Some schoolgirls, teachers and local KI spoke on the importance of behaviours regarding the misuse of toilets, as students are “careless” (Female teacher, age 39) and often urinate or defecate outside of the pits. “We must empower people who do these things with very specific rules regarding use and force them to adhere to them” (Male religious representative, age 56). Local KIs also shared that it was difficult to engage students in the cleaning task: “We try to mobilize students to clean, but it lasts shortly. Since the cleaning is often done weekly, the toilets quickly become dirty” (Male school staff, age 55). Mothers insisted cleaning should be done more regularly. There was no unanimous opinion on who should clean the toilets nor how often. One teacher proposed organising a contest to motivate students to clean. National KIs did not bring forward any particular solution in their experience.

### Theme 2. Privacy and safety

After water and cleanliness, respondents – particularly mothers and teachers – expressed worries about toilet structures, noting that the toilets featured in the pictures, at one’s own school or in schools generally are often “deteriorated”. As one mother said, “The walls are undone, the doors are spoiled (…). If you go in there to fix yourself and the wind blows, the walls will fall (on you)” (Mother, age 32). Some teachers and local KIs described how school latrines often lack doors and locks, either because they were broken or not a part of the original toilet design. For schoolgirls, safety was less emphasized and privacy concerns featured more prominently, particularly as privacy linked to menstruation. Although toilets in their schools had doors and locks, schoolgirls did not feel that they could close them well, as one girl said, “It's a little hard to manage (your period) in the bathroom, you’re not safe there because someone can open the door at any time” (Schoolgirl, age 18). Girls in both schools described how they routinely return home when they have their periods, and prefer to change and wash at home where privacy is more assured.

KIs were less forthcoming about issues of privacy and safety, noting that although both issues are important, they “don’t know who is responsible for rehabilitating toilets” (Female primary school teacher, age 41) and that “there is no maintenance plan” (Male health district informant, age 36). National KIs agreed toilets often lack maintenance, but did not give recommendations based on their experience. One local KI suggested that municipalities should be involved in monitoring for sustainability of solutions, while another national KI added that it becomes very costly to preserve interventions long-term due to the need for consistent follow-up to continue engagement.

### Theme 3. Awareness and menstrual hygiene education

KIs were most insistent on the need to raise awareness on both general hygienic behaviours and those related to menstruation. “We can’t speak about WASH without education on menstrual hygiene” (Female menstrual hygiene product informant, age 42). Teachers commented on how they did not receive any training on Menstrual Health and Hygiene (MHH) and how they would like to receive guidance in order to better help students and to learn how to talk about the subject. Some teachers and national KIs specified that the subject should be included in the schools’ curriculum. Mothers and schoolgirls also said they would like to participate in trainings on menstruation and proposed many questions to discuss throughout trainings.

### Theme 4. Sex-division

Gender division of toilets was preferred by all participants when asked, but less often brought up independently of probing. Teachers confirmed how most schools do not have sex divided toilets, or, as teachers and students described, toilets may be marked as sex divided, but the separation was not followed in practice. “Men use women's toilets, women use men's toilets and you don't know where to go to change. You can go in, and a boy can come and surprise you” (Schoolgirl, age 16). Another girl described how this would be a “disgrace”, because “the boy would tell everyone what he saw her doing” (Schoolgirl, age 16). Almost all girls shared feelings of “shame”, “fear” and “humiliation” if anyone discovered they were menstruating. Some words used by teachers to describe how they felt about the girls’ experience were “suffering”, “pity”, “compassion”, “sadness” and “embarrassment”. Most participants agreed that teachers generally do not react when girls are seen with blood stains or when they are mocked for it, and teachers simply send girls home to change. One teacher said, “I pretended I hadn't seen it [that she had a stain], because if she had known that I had seen, she would have been more ashamed and would not have come to class again” (Male teacher, age 33). Schoolgirls specified that if the teacher was a woman, “she can give you advice” (Schoolgirl, age 19) or give them a cloth or a scarf to cover themselves. Another girl explained that “If he's a good teacher, he signals you to tell you, and sends you home to change. But if it's a different person, he'll call you out in front of your classmates and blame you. Don’t you know that you're on your period? Why can't you protect yourself before coming?” (Schoolgirl, age 16). Similar distinctions were made by girls in other FGDs and IDIs.

### Theme 5. Location of toilets

Some teachers described how the geographic location of toilets was important in the sense that toilets should be far from the main school complex, for the smell not to bother the students while they are in class. Schoolgirls agreed when probed, but were concerned about being seen going to toilets multiple times in a day. One girl proposed that the toilets for girls should be far away from those for boys “because some students are rude and will try to come inside (when you are in the toilet)” (Schoolgirl, age 18), and another student said that they should not be near the classes because “the smell will prevent the students from being comfortable” (Schoolgirl, age 16). KIs and parents did not raise this issue.

### Respondent driven recommendations to develop female-friendly toilets

When asked for suggestions on what could be done to make toilets more female friendly, schoolgirls consistently requested kettles, mothers and schoolgirls highlighted privacy features (doors and locks that function) and cleaning products (soap, bleach, gloves and brooms), teachers insisted on water access and KIs called for increased budgets for school sanitation and hygiene. Schoolgirls said that plastic kettles or any mechanism that could transport water is “necessary”, “important” and would allow them to use the toilets “without fear” of people finding out they are on their period. Girls also asked for more availability of sanitary products, since their current options are disposable pads, which are prohibitively expensive, or old cloths, which are unreliable and uncomfortable. For a comprehensive breakdown of interventions suggested by participants, classified by cost and required frequency of use or installation, see **Table 3**.

**Table 3:** Recommended interventions by cost and frequency

		Frequency	
		**Low**	**High**
COST	Low	Buckets, brooms, doors with locks, reusable MHM products*	Soap and bleach, kettles, gloves, analgesics
	High	Structural improvements (foundation, roof) , changing room or infirmary	Disposable MHM products

MHM – menstrual hygiene management

*Reusable MHM products can initially be more costly, but are considered low-cost when compared long-term to disposable options.

## DISCUSSION

Our study suggests that little progress in school WASH coverage has taken place since 2013 when a case study on MHH in Burkina Faso was published in the grey literature [41]. No school in our sample met the minimum standards for WASH in schools [14], and toilets lacked a majority of the female-friendly features described in the literature. Students, and particularly schoolgirls, continue to suffer from high pupil-to-toilet ratios, lack of sex-division for toilets, absence of water access and poor cleanliness in most schools. Our results refute previous reports regarding the availability of handwashing facilities and soap [41]. While other research has found that 60% of schools in Burkina Faso have hygiene-related materials [41], no school in our study had soap available nor a functioning handwashing station (beyond a main water source, if present at all).

Robust literature has shown that local practices deserve attention when designing toilets and toilet maintenance approaches, and that intended users need to be involved in decision-making [1,4,42-47], yet girls and women are rarely included in design and development [3,48]. The way girls and women perceive and experience interventions may also widely affect intervention outcomes [49], providing yet another reason why female inclusion is crucial to fulfilling water and sanitation rights, instead of simply providing a toilet [50]. Our participants proposed several interventions that could lead the way toward a female-friendly toilet: the provision of kettles (or another form of water transport), improvement of doors and locks, and the supply of cleaning materials. Some of these interventions are notably inexpensive. For example, provision of kettles or water containers for menstrual hygiene has been reported to cost four dollars per school per year [51]. While the benefits of similar interventions have already been demonstrated [52,53] and much progress has been made in recent years [54], evidence on how to implement overarching WASH and MHH programmes effectively in different contexts remains insufficient [55,56]. More research amplifying girls’ voices is needed to develop such programmes [57].

Several aspects of our study speak to broader challenges related to sustainability. A number of facilities and improvements (eg, water sources, handwashing containers and locks) introduced in the past were now broken or unusable, which supports the need to understand and consider maintenance of installations and behaviour adherence among beneficiaries as described in the literature [58]. While maintenance and operation is considered essential [44], there is little information on how to best establish and manage these processes or how to budget for these costs on a long-term basis [59]. Many school WASH programs lack financial planning [60]. Understanding the costs of interventions, to define an appropriate budget, remains a research priority [54,61], given that there are few studies that share the costs of WASH interventions and their maintenance over time [59] and even fewer peer-reviewed studies sharing the costs of MHH interventions in particular [51,62,63]. In addition, research has shown that costs may vary extensively from country to country [59], as well as region to region within the same country [51]. Studies have also indicated the need for different institutions to be involved in management [64], with one paper describing how schools receiving external financial support from non-governmental organisations achieved better outcomes in WASH facilities [65]. However, international and private sector funding may be unreliable or controversial [59,66] and, as our KIs pointed out, the lack of clear responsibilities among multiple actors in provision, maintenance and improvement of facilities may result in their neglect. The WASH in Schools Monitoring Package developed by the United Nations International Children’s Emergency Fund also requires clear assignment of responsibilities to ensure operation and maintenance of facilities [67].

In terms of limitations, while this study provides a detailed picture of the state of school sanitation facilities in the Nouna region, we cannot assess whether or to what extent experiences can be extrapolated to similar contexts of Burkina Faso or other settings. Nevertheless, the region does not differ particularly from other regions in Burkina Faso, so a similar situation could be expected in other rural areas. Data collection also encountered delays that resulted in interviews taking place closer to the exam session, which could have limited girls’ availability and concentration. We did not, however, sense a desire for shorter interview times among schoolgirls nor did we experience a lack of volunteers to participate.

With regard to photo-elicitation, a recent study in Kenya explored pregnant and postpartum women’s experiences of water insecurity [68]. Beyond this, we are not aware of published literature applying this method in the field of WASH. Our results align with previous research, which found that photo-elicitation can yield additional depth in participants’ perceptions [69] and can facilitate verbalisation among adolescents [70]. Furthermore, this technique allowed us to pinpoint specific female-friendly features that girls desire in toilets, and we felt that the photos kept participants engaged despite the duration of IDIs and FGDs. We also sensed a change in power dynamics, with adolescents taking a more active position not only regarding the interview process itself but also in proposing and discussing different potential interventions when photos were used to guide the conversation. Photos also allowed teachers to discuss toilets more readily. In contrast, in our experience, photo-elicitation did not enrich KI interviews, as they seemed relatively uninterested in the photos, sharing their opinions and professional experience regardless of the images presented. We therefore recommend photo-elicitation as a means to approach difficult topics or to spark openness among participants who may not otherwise feel comfortable speaking freely.

## CONCLUSION

Identifying schoolgirls’ priorities and needs in different contexts is crucial to develop an appropriate plan of action that can ensure female-friendly toilets in schools are installed and maintained, especially when resources are limited. Women and girls’ leadership in informing solutions and initiatives must be expanded and their voices and recommendations must be represented in the literature.

Photo-elicitation can bring multiple advantages in the field of gender-sensitive sanitation in schools, particularly while undertaking interviews with teachers or lengthy discussions with adolescents. Further research should aim at designing and delivering high-quality and comprehensive interventions that may furnish further evidence-based data on how to best target the different features of female-friendly toilets and maintain outcomes over time.

## Additional material


Online Supplementary Document
