# Supplementary material for: Female-friendly toilets in schools in Burkina Faso: A mixed-methods study using photo-elicitation
Source: J Glob Health. 2022 Sep 8;12:04057. doi: 10.7189/jogh.12.04057 (PMC9454237; doi:10.7189/jogh.12.04057)
Supplement: Online Supplementary Document [file jogh-12-04057-s001.pdf]

<u>ONLINE SUPPLEMENTARY DOCUMENT</u>

Index

Appendix 1: COREQ checklist – Page 2

Appendix 2a: Written manual for data collectors (French version) – Page 7

Appendix 2b: Written manual for data collectors (English version) – Page 24

Appendix 3: School observation sheet – Page 39

Appendix 4: School assessment additional data – Page 41

Appendix 1: COREQ checklist

No	Item	Description	Reported on
		**omain 1: Research team and reflexivity**	
**1**	Interviewer/facilitator	*Which author/s conducted the IDI or FGD?*	Appendix 1
**2**	Credentials	*What were the researcher's credentials?*	Page 3
**3**	Occupation	*What was their occupation at the time of the study?*	Appendix 1
**4**	Gender	*Was the researcher male or female?*	Appendix 1
**5**	Experience and training	*What experience or training did the researcher have?*	Page 3
**6**	Relationship established	*Was a relationship established prior to the study?*	Appendix 1
**7**	Participant knowledge	*What did the participants know about the researcher?*	Appendix 1
**8**	Interviewer characteristics	*What characteristics were reported about the interviewer/facilitator?*	Appendix 1
		**Domain 2: Study design**	
**9**	Methodological orientation and Theory	*What methodological orientation was stated to underpin the study?*	Page 3-4
**10**	Sampling	*How were participants selected?*	Page 3
**11**	Method of approach	*How were participants approached?*	Page 4
**12**	Sample size	*How many participants were in the study?*	Table 1
**13**	Non-participation	*How many people refused to participate or dropped out?*	Appendix 1
**14**	Data collection setting	*Where was the data collected?*	Page 3-4
**15**	Presence of non-participants	*Was anyone else present besides participants and researchers?*	Appendix 1
**16**	Description of sample	*What are the important characteristics of the sample?*	Table 1
**17**	Interview guides	*Were questions, prompts, guides provided by the authors? Was it pilot tested?*	Page 3
**18**	Repeat interviews	*Were repeat interviews carried out? If yes, how many?*	Appendix 1
**19**	Audio/visual recording	*Did the research use audio/visual recording of data?*	Page 4
**20**	Field notes	*Were field notes made during/after the IDIs or FGDs?*	Appendix 1
**21**	Duration	*What was the duration of the IDIs or FGDs?*	Page 4
**22**	Data saturation	*Was data saturation discussed?*	Page 5-8
**23**	Transcripts returned	*Were transcripts returned to participants for comment/correction?*	Appendix 1
		**Domain 3: Analysis and findings**	
**24**	Number of data coders	*How many data coders coded the data?*	Page 4
**25**	Coding tree	*Did authors provide a description of the coding tree?*	Pages 6-8
**26**	Derivation of themes	*Were themes identified in advance or derived from data?*	Page 4

**27**	Software	*What software was used to manage the data?*	Page 4
**28**	Participant checking	*Did participants provide feedback on the findings?*	Appendix 1
**29**	Quotations presented	*Were participant quotations presented to illustrate the themes / findings? Was each quotation identified?*	Pages 5-8
**30**	Data and findings consistent	*Was there consistency between the data and the findings?*	Page 5-8
**31**	Clarity of major themes	*Were major themes clearly presented?*	Pages 6-8
**32**	Clarity of minor themes	*Is there a description of diverse cases or discussion of minor themes?*	Pages 6-8

Adapted from: Tong A, Sainsbury P, Craig J. Consolidated criteria for reporting qualitative research (COREQ): a 32-item checklist for interviews and focus groups. Int J Qual Health Care. 2007:19 (6);349-357.

Domain 1: Research team and reflexivity

1. **Interviewer/Facilitator:** Méma Sanogo, Aoua Drabo, Clarisse L. Sow, Djénèba Kanazoe, Moussa Ouedraogo and Jacouba Moukoro are included in "Acknowledgements", according to the authorship criteria by the International Committee of Medical Journal Editor. Jean-Louis Koulidiati is a co-author of this paper.

Table 1. Details of interviewers/facilitators

	Gender	Credentials	Experience
MS	Female	High school, college attendance	Extensive research experience, some in qualitative research
AD	Female	High school, college attendance	Multiple qualitative research studies
CLS	Female	Master in Political Science	Extensive research experience, some in qualitative research
DK	Female	High school	No experience in data collection
MO	Male	Master in Sociology	Extensive qualitative research experience
JM	Male	Pedagogy degree	Multiple qualitative research studies
JLK	Male	Doctor of Medicine, PhD in Health Economics	Extensive research experience, some in qualitative research

2. **Credentials:** See Table 1.
3. **Occupation:** All interviewers were contracted by or otherwise employed within the Nouna Health Research Centre (NHRC), except for Jean-Louis Koulidiati who was a Burkinabe researcher at the Heidelberg Institute of Global Health (HIGH).
4. **Gender:** See Table 1.
5. **Experience and training:** See Table 1. All interviewers (except Jean-Louis Koulidiati) participated in the data collector training, designed for our study by Teresa Buitrago García and Shannon McMahon, prior to the start of data collection. Jean-Louis Koulidiati joined our team later to serve as our representative in Ouagadougo (Burkina Faso), where several national-level key informants (KIs) were based. He was provided with all the materials from the initial training in Nouna, which were discussed with Teresa Buitrago García.
6. **Relationship established:** There was no personal relationship between interviewers and participants prior to the start of data collection. However, participants knew the institution they represented (NHRC), as it is well known and highly regarded in the town.

7. **Participant knowledge of the interviewer:** Interviewers introduced themselves as NHRC members upon arrival at the schools and explained that the study aimed at learning more about the challenges girls face at school, regarding menstruation and sanitation facilities. No personal goals were shared.
8. **Interviewer characteristics:** Facilitators reported no personal biases.

Domain 2: Study design

9. **Methodological orientation and theory:** This study was guided by the tenets of human-centered design in the sense that our ultimate goal is to develop an intervention related to menstrual hygiene management that is responsive to the needs of end users (menstruating adolescents) and program implementers (individuals at household, community or local levels who might enact any future intervention), and which is sensitive to the general context (the state of existing toilets, and the nature of existing discourse on hygiene management).

   Analysis began in the field via group debriefings, and formal analysis of transcripts (guided by thematic analysis) was performed on all transcripts by the two lead authors (Teresa Buitrago García, N Hélène Sawadogo) who exchanged insights and compared codes. In instances when there was disagreement about a code or questions related to coding, the lead authors sought insights from a senior author (Shannon McMahon).
10. **Sampling:** Purposive sampling.
11. **Method of approach:** Face-to-face.
12. **Sample size:** See "Table 1" in the main document.
13. **Non-participation:** We experienced neither drop-outs nor refusals.
14. **Setting of data collection:** Schoolgirls, mothers and teachers were interviewed at their schools. KIs were interviewed at their workplaces.
15. **Presence of non-participants:** Only interviewers and participants were present in the room at the time of IDIs and FGDs.
16. **Description of the sample:** See "Table 1" in the main document.
17. **Interview guide:** We created interview guides for each of the respondent groups, including prompts in all sections. All tools were piloted in a school that was not included in the later study, and a debriefing was done among interviewers, as well as between the coordinators at NHRC and HIGH, to discuss findings and necessary edits.
18. **Repeat interviews:** We did not repeat any interviews.
19. **Audio/visual recording:** All interviews were audio recorded, with informed consent from all participants.
20. **Field notes:** Notes were taken during IDIs (by interviewer) and FGDs (by note-taker). N Hélène Sawadogo took notes during daily debriefings throughout data collection, sharing and discussing findings via phone calls every 2-3 days with Teresa Buitrago García, who also took notes throughout the process.
21. **Duration:**
    - FGDs with schoolgirls: median 100 minutes (range 85 to 162).
    - IDIs with schoolgirls: median 55 minutes (range 35 to 110).
    - FGDs with mothers: median 155 minutes (range 111 to 166).
    - IDIs with teachers: median 86 minutes (range 77 to 102).

- IDIs with local KIs: median 72 minutes (range 55 to 93). - IDIs with national KIs: median 55 (range 46 to 60).

22. **Data saturation:** Data saturation was discussed during debriefings. While some minor themes appeared, especially in interviews with KIs who would expand on their personal experiences, based on their individual experience, major themes began to repeat throughout interviews with schoolgirls, mothers and teachers, which prompted the team to conclude data collection.
23. **Transcripts returned:** Transcripts were not returned to participants for comment/correction.

Domain 3: Analysis and findings

24. **Number of data coders:** Two.
25. **Description of the coding tree:**

Table 2. Codes and coding across respondent groups

	**Main codes**		**Themes**	**Major themes**
**Schoolgirls**	Water= kettles<br>Clean<br>Soap<br>Doors<br>Reliable locks<br>MHM products<br>Sink	Sex-division<br>Good use of toilets<br>Location<br>Smell<br>Changing room<br>Shower<br>Roof	Water access<br>Cleanliness<br>Privacy<br>Safety<br>Hygiene education<br>MHH education<br>Awareness<br>MHM products<br>Sex-division<br>School<br>curriculum<br>Organisation<br>barriers Location<br>Other FF features	Water access<br>Privacy and safety<br>Awareness<br>Sex-division<br>Location
**Mothers**	Water = kettles<br>Soap<br>Clean<br>Cleaning materials<br>Safety<br>Doors<br>Locks	MHM products<br>Well-built<br>Good use of toilets<br>Sex-division<br>A place to throw pads		
**Teachers**	Water = kettles<br>Privacy =doors<br>Locks<br>Clean<br>Soap<br>Cleaning materials<br>Sink	School curriculum<br>Awareness<br>MHM products<br>Location<br>Sex-division<br>Other toiletries<br>Roof		
**Local key informants**	Water=kettles<br>Sex-division<br>Unclear responsibility<br>Clean<br>Soap<br>Cleaning materials<br>Cleaning system	Doors<br>Locks<br>Awareness and training<br>MHM products<br>Insufficient nº of toilets<br>Place to leave things<br>Negative privacy<br>Roof		

**National key informants**	MHH education<br>School curriculum<br>Water<br>Sex-division<br>Cleanliness<br>Soap	MHM products<br>Place to leave things<br>Good use of toilets<br>Changing room<br>A place to throw pads<br>Disability access		

MHH – Menstrual health and hygiene; MHM – Menstrual hygiene management; FF – Female-friendly.
Boxes in grey show the codes that were more frequently used in interviews with each respondent group.

26. **Derivation of themes:** After reading and discussing the transcripts, we generated the initial codes, which we analysed further to identify arising themes. These themes were consistent with the themes discussed during debriefings throughout data collection. On an additional step, we focused on the connections between themes, to determine the major themes, as they appear in the main document.
27. **Software:** NVivo 12.
28. **Participant checking:** There was no participant checking.
29. **Quotations presented:** We identified all quotations with respondent group, gender and age. In the case of KIs, we provided their age and field of expertise.
30. **Data findings consistent:** Our analysis was consistent with the data derived from both quantitative observations and qualitative interviews. Triangulation of data among respondent groups did not reveal any inconsistencies either. Themes arising from photo-elicitation and the rest of the interview also aligned.
31. **Clarity of major themes:** We identified the major themes as separate sections throughout the "Results" section, in the main document.
32. **Clarity of minor themes:** We presented minor themes in relation to major themes or in relation to the respondent group, when the theme was mostly exclusive to one set of participants.

Appendix 2a: Written manual for data collectors (French version)

Table des matières

1. INTRODUCTION 25
   1.1. Contexte de l’étude 8
   1.2. Les objectifs de l'étude 8

2. L’ÉTUDE 3
   2.1. Site de l’étude 3
   2.2. Modèle de l´étude 25
   2.3. Organisation de l’enquête 6
   2.4. Les instruments de l´étude 7
   2.5. Discussion de groupe 8
   2.6. Entretien individuel 9
   2.7. Votre rôle comme enquêteur 9
   2.8. Surveillance de l’étude 10

3. LES PRODUITS SANITAIRES 10
4. PROCÉDURES GÉNÉRALES 11
4.1. Critères d´inclusion et d´exclusion 12
4.2. Le consentement éclairé 12
Personne lettrée 13
Personne illettrée 13
5. COLLECTE DE DONNEES QUALITATIVES 13
4.1. Règles générales pour la collecte de données qualitatives 13
4.2. Evènements défavorables 16
6. L'UTILISATION DU DICTAPHONE.. 16

1. INTRODUCTION

1.1. Contexte de l'étude

L'apparition de la menstruation peut être une période difficile pour beaucoup de filles dans le monde entier et peut être un défi pour leur développement et bien-être physique, mental et social. Plusieurs fois, elles ne savent pas ce qui leur arrive ou considèrent cela comme une maladie. Elles se sentent souvent seuls et n'ont personne à qui parler. Le sujet est tabou et de nombreuses règles sociales peuvent conduire à leur isolement. Elles peuvent aussi souffrir d'autres problèmes de santé qui résultent d'une hygiène menstruelle inappropriée. Dans d'autres pays, des études ont montré que les filles peuvent également recourir à des pratiques dangereuses pour se procurer des produits sanitaires, souvent de difficile accès.

Les filles commencent souvent à manquer l'école ou à l'abandonner complètement. Certaines des raisons évoquées sont le manque d'installations à l'école, les camarades de classe qui les taquinent, la difficulté à se concentrer à l'école, des problèmes d'estime de soi, etc. C'est pour ces raisons, entre autres, que la gestion de l'hygiène menstruelle fait l'objet d'une attention croissante ces dernières années, notamment dans la mesure où les secteurs de la santé et de l'éducation cherchent à réduire les disparités en matière d'éducation, à promouvoir l'égalité des sexes et à autonomiser les femmes et les filles.

Nous aimerions comprendre quelles en sont les raisons sous différents angles afin que les institutions impliquées puissent développer ou améliorer des programmes qui les aideront. Chaque pays et chaque région sont très différents, de même manière que les personnes qui y vivent, et il y a souvent de nombreuses croyances socioculturelles et pratiques régionales qui ont une grande influence que nous voudrions examiner. Tous ces aspects seront explorés lors des entretiens.

1.2. Les objectifs d'étude

Ce travail cherche à comprendre la situation actuelle en ce qui concerne la menstruation et à identifier les défis et les facilitateurs de la gestion de l'hygiène menstruelle à Nouna (Burkina Faso), qu'il s'agisse de contraintes économiques, socioculturelles et / ou infrastructurelles. Cette phase formative de recherche testera également, à très petite échelle, la faisabilité et l'acceptabilité de divers produits. Les résultats de cette recherche serviront de base à une deuxième phase de recherche en 2019/20, qui examinera les résultats associés à la fourniture de produits de gestion de l'hygiène menstruelle à plus grande échelle.

2. L'ÉTUDE

2.1. Site de l'étude

Cette étude sera menée dans le site de surveillance démographique et de santé (SSDS) de Nouna, dans la province de Kossi. Il se déroulera au Centre de Recherche en Santé de Nouna (CRSN). Une grande partie de cette zone est rurale et 30% de la population vit dans la ville de Nouna. Selon les données les plus récentes (2015), environ 107 000 personnes vivaient dans la zone de recensement du CRSN. Selon une étude récente sur la «Santé des Adolescents» de l'Institut de Santé Globale de Heidelberg sur la mutilation génitale féminine chez Nouna SSDS, les filles entre 12 et 20 ans souffrent d'un grave manque de connaissances sur la menstruation. Plus de 80% des femmes rurales au Burkina Faso utilisent des vieux tissus pour gérer leurs règles.

2.2. Modèle de l´étude

L’étude a été développée en coopération entre le CRSN et l'Institut de Santé Globale de l'Université de Heidelberg. Il y aura deux phases de cette étude.

Lot de travail	Type de collecte des données qualitatives	Source de données
1. Phase de génération de connaissances: attitudes, expériences et pratiques actuelles liées à la menstruation et à la gestion de l’hygiène menstruelle	• Observations<br>• Discussions de groupe<br>• Entretiens individuels semi-structurés	Observations dans les écoles<br>Discussions de groupe avec les écolières et les mères (divisés par le type de répondant)<br>Entretien individuels avec les enseignants et les informateurs clés (représentants impliqués dans les politiques ou interventions en matière d'eau et d'hygiène dans les écoles, de l'*UNICEF*, des organisations non gouvernementales (ONG), du Ministère de la Santé ou de l'Éducation et / ou des membres du conseil scolaire)
2. Phase de *minitesting* : faisabilité et acceptabilité des produits	• Entretiens individuels semi-structurées<br>• Étude de journal	Entretien individuels avec les écolières pre et postdistribution du produit<br>Étude de journal avec les écolières

a. Phase de perspectives générales

Observations: Nous avons déjà collecté des informations sur les écoles secondaires de Nouna, notamment le nombre d’enseignantes, le nombre de filles qui ont plus de 12 ans, l’accès à l’eau, les latrines, la présence de lieux d’échange, etc. Il s’agit d’une étape importante pour évaluer si la distribution d’un produit aux écolières est réalisable (à petite échelle ou à plus grande échelle pour une phase ultérieure de ce travail).

Discussion de groupe: jusqu’à 15 discussions de groupe avec 6-8 participants par groupe seront organisées, comprenant des discussions avec les écolières et les mères, respectivement. Les questions porteront sur les croyances en relation à la menstruation, leur expérience personnelle, les connaissances sur les produits de gestion de l’hygiène menstruelle, les principaux obstacles à l'accès aux produits, la perception locale des produits et la faisabilité et l'acceptabilité des produits potentiels. En outre, nous visons à explorer des facteurs sociétaux tels que les tabous ou les opinions concernant la menstruation et produits sanitaires. Veuillez lire attentivement les questionnaires avant le jour correspondant de votre formation.

Entretiens individuels: Des entretiens semi-structurés comprenant jusqu'à 10 enseignants et jusqu'à 15 informateurs clés (tels que des représentants de l'*UNICEF*, des organisations non gouvernementales, du Ministère de la Santé ou de l'Éducation et / ou des membres du conseil scolaire) seront conduit. Ils incluront des questions qui se concentrent sur les normes existantes et sur la manière dont différentes interventions de gestion de l’hygiène menstruelle pourraient être intégrées dans les routines existantes. Nous allons essayer de comprendre: Comment sont l'hygiène et particulièrement l'hygiène féminine généralement dans les écoles? Qui pourrait être le mieux placé pour guider les écolières en termes de la menstruation et de l’hygiène menstruelle? Comment cette conversation devrait-elle avoir lieu et que devrait comprendre ou exclure la conversation? Qu'est-ce qui est le plus réalisable en termes d'amélioration de la gestion de l’hygiène menstruelle dans les

écoles, que ce soit sur le type du produit (serviettes par rapport à coupes menstruelles ou sousvêtements rembourrés) et en termes de distribution du produit? Veuillez lire attentivement les questionnaires avant le jour correspondant de votre formation.

Entretiens individuels predistribution avec des écolières: Le groupe d'écolières sélectionné de les groupes de discussion participera à des entretien individuels semi-structurés avant de recevoir les produits sanitaires. Ces entretiens examineront leurs perspectives générales sur la menstruation et leur expérience et connaissances. Il y a également des questions sur la gestion de l'hygiène menstruelle (à l'école, à la maison, les produits sanitaires qu'elles connaissent, etc.) et sur leurs recommandations et leurs besoins. Ces entretiens fourniront un environnement plus privé où les filles pourront partager plus sur les thèmes discutés lors des discussions de groupe.

b. Phase de *mini-testing*

Nous présenterons les produits dans les discussions de groupe avec les écolières et les mères. Un groupe choisi d'écolières recevra des produits sanitaires qui seront testés pendant une période de deux mois. Les alternatives seront présentées dans ce manuel dans le chapitre « 2.7. Les produits sanitaires ». Chaque participante pourra choisir et essayer un seul produit. Jusqu'à 30 écolières seront sélectionnées pour cette phase.

Entretiens individuels avec les écolières postdistribution : Après deux mois d'utilisation des produits, un autre entretien individuel semi-structuré sera consacré aux détails de leur expérience avec les produits sanitaires. Au cours de ces entretiens, nous voulons comprendre les opinions des filles sur différents aspects du processus de distribution, les connaissances qu'elles aimeraient acquérir, le type de produits qu'elles préfèrent, les difficultés qu'elles rencontrent pour les utiliser et le soutien qu'elles ont reçu (À qui aimeraient-elles parler? Quelles questions avaient-elles? Qui devrait leur donner les produits?, etc). Ce sera une interview dans laquelle nous leur demanderons de concevoir leur programme idéal pour la gestion de l'hygiène menstruelle, en précisant les changements dans la façon dont nous l'avons fait. Nous explorerons en outre leur expérience avec le journal et discuterons des questions qu'ill contient.

Journal: Les participants recevront un journal contenant des questions sur leur expérience du produit, qui sera discuté lors de l'entretien individuel postdistribution. Il y a certaines idées ou pensées qu'elles pourraient oublier au cours des 2 mois d'utilisation. Le temps change la qualité et l'intensité de nos souvenirs. Les choses qui semblaient bonnes ou mauvaises au début peuvent sembler différentes après un certain temps, quand nous nous y sommes habitués. Le journal leur permet d'écrire leurs réflexions sur les produits en même temps qu'elles les découvrent. Veuillez lire attentivement les questionnaires et le journal avant le jour correspondant de votre formation. Le tableau suivant est un résumé de la taille de l'échantillon en fonction de ces groupes de participants décrits.

Type d'entretien	Quantité d'entretiens	Quantité de participants	Taille max. de l'échantillon
Discussion de groupe avec mères	7-8 groupes	6-8 mères/groupe	64
Discussion de groupe avec filles	7 groupes	6-8 filles/groupe	56

Entretien individuel avec enseignants	≤10 entretiens	≤10 enseignants	10
Entretien individuel avec informateurs clés	≤15 entretiens	≤ 15 informateurs clés	15
Entretien individuel predistribution avec filles	≤30 entretiens	≤30 écolières	30
Entretien individuel postdistribution avec filles	≤30 entretiens	≤ 30 écolières	30

Ce sont les chiffres établis en accord avec le Comité Éthique de Nouna et Heidelberg. Par conséquent, ces limites doivent être respectés. D'autre part, le nombre de participants peut être inférieur si cela est justifié.

2.3. Organisation de l'enquête

L'ordre réel des entretiens et groupes de discussion sera un peu différent en raison des questions pratiques et logistiques.

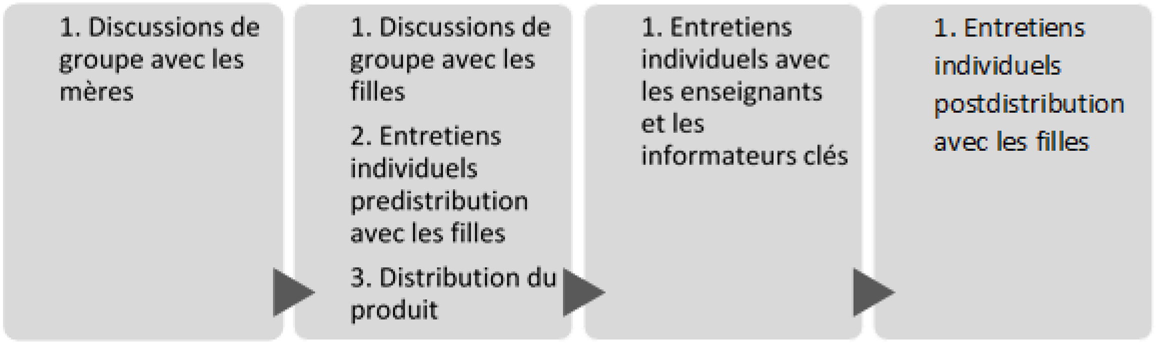

Au cours de la première semaine, nous aurions des discussions de groupe avec les mères. Cela nous permettra de signer les formulaires de consentement, dans le cas des filles de moins de 18 ans. Cela nous fera également gagner du temps, car nous n'aurons besoin de réunir les mères qu'une seule fois.

Une fois ces discussions de groupe terminées, nous les mènerons avec les filles. Habituellement, une discussion de groupe par jour suivie de 2-3 entretiens individuels avec les filles sélectionnées. Cela dépend du nombre d'enquêteurs disponibles. Il est important que les discussions de groupe et les entretiens se déroulent le même jour, car nous souhaitons que le choix du produit soit aussi neutre que possible (une étudiante décide par elle-même). Immédiatement après l'entretien predistribution, nous leur donnerons le produit sanitaire choisi. Une fois toutes les discussions de groupe terminées, nous pourrons poursuivre les entretiens individuels avec les enseignants et les informateurs clés. Enfin, deux mois après avoir donné le produit sanitaire aux filles, nous réaliserons les entretiens individuels postdistribution. Le calendrier du projet peut varier en fonction de nombreux facteurs. Notre objectif n'est pas de faire beaucoup d'entretiens par jour pour finir rapidement. Les enquêteurs doivent être conscients de leurs compétences et limitations et planifier en conséquence. C'est normal d'être fatigué après plusieurs entretiens et notre capacité à mener la conversation, à écouter activement et à reconnaître les points qui méritent d'être explorés est un processus très épuisant. C'est pourquoi faire trop d'entretiens en une même journée peut résulter dans des données de mauvaise qualité. La chronologie suivante est simplement une estimation du temps que nous pensons chacune des phases va prendre:

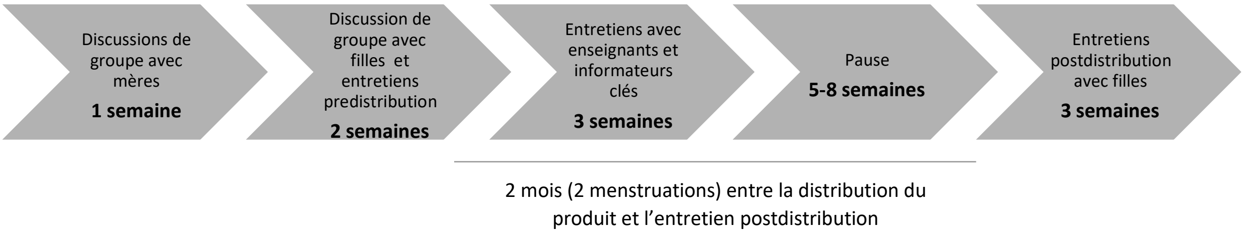

2.4. Les instruments de l´étude

Les instruments de l'étude seront adaptés pendant la formation des enquêtrices et le pilotage, et pourront ensuite être adaptés de nouveau en fonction des résultats des premiers groupes de discussion et des entretiens individuels. Les questions ne sont jamais résolues, nous pouvons toujours apporter des modifications, si nécessaire. La modification des questionnaires est essentielle pour la qualité des données qualitatives. Les points soulignés ensuite ne sont également que quelques-uns des sujets à explorer. Au cours des discussions de groupe et des entretiens, d'autres sujets abordés par les participants pourraient devenir significatifs pour l'étude et vous êtes encouragés à les poursuivre.

a. Groupes de discussion avec les mères

Objectif: Étudier le rôle des mères dans la communication des connaissances, des informations, des attitudes et des pratiques concernant la menstruation de leurs filles; Comprendre l'opinion des mères sur les difficultés que rencontrent les filles dans les écoles.

Domaines d'enquête:

(1) Les connaissances, perceptions et comportements des femmes;
(2) Le soutien des mères aux filles et leur compréhension de leurs expériences; (3) Compréhension par les mères des expériences des filles à l'école; (4) Recommandations.

b. Discussions de groupe avec les filles

Objectif: Étudier et identifier les expériences, les défis et les besoins d'une fille typique.

Domaines d'enquête:

(1) Caractéristiques des installations scolaires actuelles et idéales;
(2) Changements de vie et normes sociales;
(3) Expériences et défis liés à la menstruation;
(4) Recommandations pour améliorer les expériences des filles.

c. Entretiens individuels predistribution avec les filles

Objectif: Le sujet de l'entretien est similaire, mais elles pourraient en partager davantage en privé. Nous parlons également plus de produits sanitaires spécifiques et de leurs besoins.

Domaines d'enquête:

(1) Connaissances et perceptions sur la menstruation et la gestion de l'hygiène menstruelle;
(2) Expérience personnelle avec la menstruation;
(3) Comportements pendant la menstruation;
(4) Recommandations.

d. Entretiens individuels postdistribution avec les filles

Objectif: Découvrir le meilleur moyen de distribuer un produit et les mesures pratiques permettant d'améliorer leur expérience.

Domaines d'enquête:

(1) Leur expérience avec le produit choisi;
(2) Leur expérience avec le journal;
(3) Création hypothétique d'un programme idéal; (4) Recommandations.

e. Entretiens individuels avec les enseignants

Objectif: Obtenir des informations sur l'école et les points de vue des enseignants sur les problèmes liés aux règles en milieu scolaire.

Domaines d'enquête:

(1) Normes sociales et comportements;
(2) Perspectives des enseignants sur la menstruation et l'hygiène menstruelle; (3) Systèmes de support pouvant être développés; (4) Recommandations.

f. Entretiens individuels avec les informateurs clés

Objectif: Identifier les systèmes existants à renforcer et les bonnes pratiques.

Domaines d'enquête:

(1) Perceptions de la population, des institutions et du gouvernement
(2) Défis liés à la menstruation et à l'hygiène menstruelle et leur expérience professionnelle (3) Programmes et campagnes déjà développés
(4) Recommandations.

2.5. Discussion de groupe

Pour une discussion de groupe, on rassemble un groupe de gens qui respecte les critères d´inclusion. Le groupe a un modérateur et une autre personne prend des notes. La discussion est enregistrée et transcrite. Dans le groupe, on veut bien écouter les participantes et leurs idées générales sur le sujet concerné. On veut comprendre l´attitude et les expériences des participantes. Dans le groupe on gagne aussi des informations sur l´environnement naturel des participantes parce qu´elles sont en groupe et on peut observer les interactions. Quelques fois on demande aux participantes d´accomplir un exercice, comme écrire des mots sur un tableau ou discuter d'images.

Le modérateur est la personne qui guide la discussion. Il essaie de mettre les participants à l´aise et de leur signaliser qu´on écoute ce qu´elles disent. Il y a un guide d'entretien avec des questions que le modérateur pose. C´est très important que les participantes soient encouragés à raconter et que les réponses aient une certaine profondeur. Il y a quelques tactiques pour recevoir des réponses suffisantes: on peut sonder en demandant une explication, un éclaircissement, une spécification, un exemple ou l´opinion personnelle. Les règles des entretiens qualitatives en général sont expliquées plus tard dans ce manuel.
La personne qui prend les notes est l´observateur du groupe. Dans un groupe de discussion ce ne sont pas seulement les réponses des participantes qui sont importantes mais aussi leur interaction. Comment réagissent les autres quand une participante explique son opinion? Sont-elles d´accord? Y

a-t-il une hiérarchie dans le groupe? L´observateur peut aussi noter quand il y a des problèmes de compréhension avec certaines questions, quand il a l´impression que les participantes sont fatiguées ou autres choses qui pouvaient être important à notifier. L´observateur aide le modérateur avec le mot de bienvenu des participantes et s'assure que l´enregistrement fonctionne bien.

2.6. Entretien individuel

Le but d´un entretien qualitatif est de gagner des informations profondes des points de vue du participant et de comprendre ses interprétations du monde. On a un guide d'entretien, mais on peut formuler les questions différemment si le participant ne les comprend pas. Le guide d'entretien assure que l'enquêteur couvre les mêmes sujets avec chaque participant. C´est important que le participant soit encouragé à raconter plus et que ses réponses ne sont pas trop courtes et superficielles. L'enquêteur peut lui demander de spécifier, d´expliquer ou de donner un exemple quand il a l´impression que la réponse n´est pas assez détaillée. On enregistre l´entretien et le transcrit après. L´enquêteur essaie de se comporter dans une manière que le participant se sent à l´aise et peut avoir confiance. On appelle cela bon rapport. Dans le meilleur cas, le participant a l´impression d´avoir plutôt une conversation qu´un entretien. Des stratégies pour développer un bon rapport sont par exemple maintenir le contact des yeux, hocher la tête pour encourager le participant, commenter la réponse quand cela est approprié et ne pas interrompre le participant.

2.7. Votre rôle comme enquêteur

Comme enquêteur vous êtes responsable de la collecte des données. Vous demanderez le consentement et conduisez les discussions de groupe avec les femmes et les filles et les entretiens individuels avec les filles, les enseignants et les informateurs clés. Votre collaboration est de la plus haute importance et sert à garantir la qualité des données rassemblées et nous aide à respecter le calendrier de l'étude.

La collecte de données d'une haute qualité est très importante pour une étude réussie. Dans le futur, les données peuvent servir à améliorer les services de santé dans la région et la situation de vie des femmes et des filles. Comme enquêteur, vous faites partie d'une équipe de recherche. Dans l'équipe, il est important que nous identifions des problèmes qui surviennent pendant la réalisation de l'enquête et de régler des problèmes liés à la collecte des données pour qu'ils ne s'accumulent pas et rendent les données inutilisables. Vous allez enregistrer les discussions de groupe et les entretiens individuels. En collectant les données, il est important que vous respectiez les participants, que vous écoutiez attentivement et que vous posez les questions de manière à ce que les participants répondent avec une profondeur suffisante.

Si vous êtes le preneur de notes/observateur, votre rôle est également indispensable. Rappelez-vous que les notes sont aussi des données! Elles sont pris même lorsqu'un dictaphone est utilisé. Vous devez noter les principaux problèmes soulevés, le langage du corps et la communication non verbale, les interactions de groupe, le langage parlé/familier, les interruptions, les personnes du groupe qui dominent la conversation, les personnes qui sont généralement silencieuses. Ces notes sont primordiales pour le débriefing, la transcription et l'analyse de données. Rassurez-vous d'avoir ces compétences à l'avance.
En tout cas, enquêteur ou preneur de notes, votre travail va être vérifié régulièrement par un coordinateur de recherche. Des bons résultats peuvent être obtenus que par un travail consciencieux. Vous pouvez toujours discuter avec ou demander l'aide d'un des coordinateurs si vous avez des problèmes ou des inquiétudes, quoi qu'elles soient. Nous espérons que vous apprendrez et acquerrez

des compétences valables dans le domaine de la recherche au cours de cette étude. N'oubliez pas que l'érudition est un processus continu et qu'en travaillant avec ardeur, on obtient les meilleurs résultats personnels et scientifiques.

2.8. Surveillance de l'étude

Cette étude est une coopération entre le CRSN et l'Institut de Santé Globale de l'Université de Heidelberg, Allemagne.

Dr. McMahon, l'investigatrice principale, mène des recherches dans les domaines de la santé maternelle, infantile et questions de santé connexes dans de nombreux pays d'Afrique Subsaharienne depuis 9 ans. Elle a mené des études sur la gestion de l'hygiène menstruelle au Kenya et sur la santé et l'autonomisation des écolières au Mali. En qualité de chef de l'unité des interventions comportementales à l'Institut Heidelberg de Santé Globale, elle a passé l'année dernière en travaillant en étroite collaboration avec le CRSN pour développer des interventions sanitaires liées à la grossesse et à la nutrition.

La Dr. McMahon travaillera en étroite collaboration avec le Dr. Ali Sié, chercheur Burkinabé, et la collègue du Dr. Sié, qui supervise les recherches qualitatives, Mme N. Hélène Sawadogo. Le Dr. Sié est le directeur du CRSN depuis 2007 et travaille au CRSN depuis 2004. Il a mené plusieurs enquêtes scientifiques sur les conditions de santé et les systèmes de santé. Mme Sawadogo est la coresponsable de la recherche qualitative sur la nutrition et la santé, qui a conduit à un partenariat solide entre elle et la Dr. McMahon.

3. LES PRODUITS SANITAIRES

Dans cette partie du manuel, nous présenterons les produits qui seront proposés aux écolières après l'entretien. Il est important que les filles choisissent librement. Leurs choix sont aussi des données. De cette manière, nous souhaitons que vous restiez le plus neutre possible lors de la présentation des produits pendant des discussions de groupe.

Pendant cette partie de la formation, vous pourrez voir les produits et en prendre connaissance. Ce sera un bon moment pour partager vos opinions, vos pensées et vos attentes concernant chacun des produits. N'hésitez pas à poser des questions à leur sujet. Tous ces produits ont été testés et peuvent être utilisés en toute sécurité.

Sous-vêtements absorbants ThinX

Les sous-vêtements ont la capacité de maximum 2 serviettes hygiéniques. Ils doivent être lavés à l'eau froide et au savon et séchés au soleil ou dans un endroit sec.

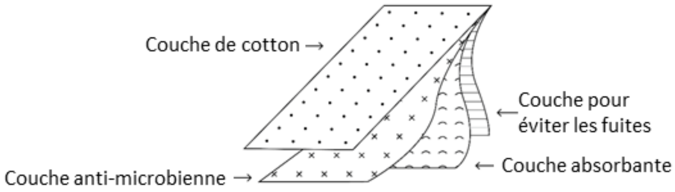

Serviettes hygiéniques réutilisables Palobdé

Les serviettes sont en coton. Chaque serviette vient avec un insert imperméable. Les serviettes doivent être changées toutes les quelques heures et lavées avec du savon après son utilisation. Elles doivent

être séchées dans un endroit sec. Elles peuvent être réutilisées pendant deux ans. Elles sont fabriquées au Burkina Faso.

Serviettes hygiéniques jetables

Elles doivent également être changées régulièrement, en fonction du flux menstruel. Il ne faut pas jeter les serviettes par terre ou dans les toilettes. Les serviettes doivent être brulées ou jetées dans des poubelles spéciales.

Coupe menstruelle

Elle doit être mise dans l'eau bouillante pendant 3-5 minutes au début et à la fin des règles, avant de la retourner à son sac. Il faut se laver les mains avec du savon avant de l'introduire et avant de la changer. La coupe doit être lavée aussi avant de la réintroduire. On doit vider la coupe chaque 12 heures environ. Si la coupe tombe dans les toilettes ou sur le sol, ne pas la réintroduire. Faites-la bouillir avant de l'utiliser à nouveau. Elle peut être réutilisée jusqu'à 10 ans.

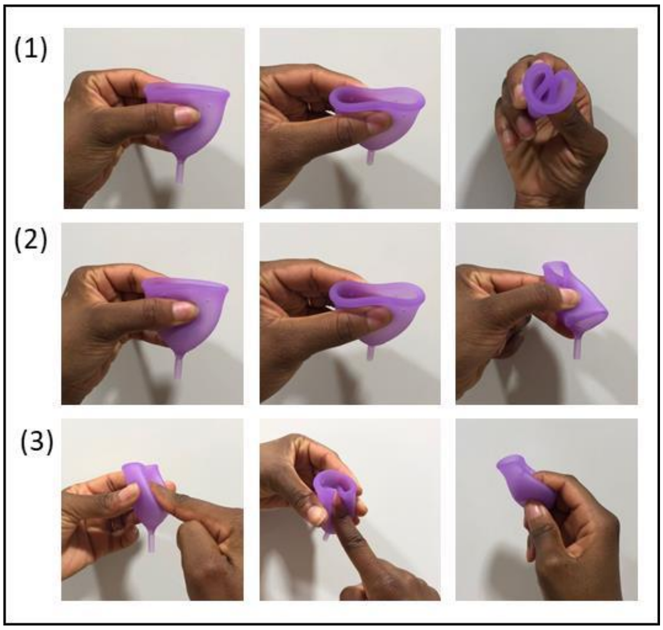

Ici, vous pouvez voir quelques-unes des façons de la plier pour l'introduire.

4. PROCEDURES GENERALES

4.1. Critères d´inclusion et d´exclusion

Quand vous êtes sur le terrain et vous avez trouvé le participant avec la liste des participants, vous vérifiez encore une fois, si le participant met les critères d´inclusion. L'ethnicité, la race, l'orientation politique, la religion et la classe ne sont pas des critères d'inclusion ou d'exclusion dans cette étude.

Pour être en mesure de refléter l'objectif ultime de l'essai ultérieur (qui évaluera l'impact de la fourniture des produits sanitaires sur la fréquentation scolaire), il est nécessaire d'inclure les filles à partir du moment de l'apparition des règles (dès l'âge de 12 ans). Étant donné que nous visons à déterminer une solution réalisable et acceptable dans le domaine de la gestion de l'hygiène

menstruelle pour les femmes dans le contexte des zones marginalisées, nous souhaitons être en mesure de fournir cette solution aux femmes dès leur naissance.

Impliquer les filles plus jeunes dans l'éducation sur l'hygiène menstruelle et dans les pratiques menstruelles sûres, grâce à la fourniture de produits sanitaires est un moyen de résoudre les difficultés et les obstacles liés à ce groupe d'âge, qui diffèrent probablement de ceux liés aux femmes plus âgées.

**Critères d´inclusion**	**Critères d´exclusion**
Âge 12 ou plus<br>Capable de donner consentement éclairé, si âgé > 17 ans<br>Disposé et capable de donner consentement éclairé et avoir un parent/tuteur disposé et capable de donner consentement éclairé, si âgé < 17 ans	Âge < 12 ans<br>Incapacité<br>Incapable de donner consentement éclairé, si âgé > 17 ans<br>Parent/tuteur refuse le consentement, si âgé < 17 ans<br>Participant refuse consentement

4.2. Le consentement éclairé

Le consentement éclairé est un consentement volontaire de participation dans la recherche. Le consentement est un processus dynamique et pas seulement une signature sur une fiche. Il continue tout au long de l'étude et il n'est jamais définitif! Afin d'obtenir un consentement éclairé, c'est essentiel que le participant comprenne parfaitement l'information donnée sur la recherche et les risques et bénéfices de la participation à l'étude. Le but est que l'individu reçoit toute information nécessaire pour arriver à une décision informée sur sa volonté de participer à l'étude ou non, sans avoir subi de pression, d'influence exagérée ou d'intimidation. Dans le cas d'écolières de moins de 18 ans, vous devrez obtenir le consentement éclairé de son parent ou de son tuteur et suivre le même processus informatif avec les deux, la fille et son tuteur/parent.

Le processus de consentement éclairé est un dialogue sur les objectives de l'étude, sa durée, les procédures, alternatives, risques et bénéfices. C'est un processus qui continue au long de l'étude : les participants peuvent retirer le consentement à tout point de l'étude. Il est votre responsabilité d'assurer que les participants comprennent toutes les informations et pour quoi ils s'inscrivent et qu'ils comprennent le concept du consentement éclairé.

La fiche du consentement éclairé est une partie importante de la procédure de consentement éclairé. Cette fiche inclut des informations sur le but de la recherche, les procédures d'enquête, des risques, potentielles bénéfices de cette recherche pour la société et des individus, le temps que l'enquête prendra, des informations de contact des investigateurs principales, une déclaration que la participation à l'étude est volontaire, le droit à confidentialité et le droit de retirer de l'étude á tout point sans conséquences.

Dans cette étude, la fiche de consentement éclairé est une fiche en papier. Elle va contenir des informations identifiants du participant, y compris leur signature ou une empreinte digitale s'il est illettré. Il est extrêmement important que vous sauvegardiez ce document et que ses contenus ne peuvent pas être vus ou lus par d'autres personnes.
Parfois, un individu ne peut pas donner un consentement éclairé. Dans ce cas, ils ne peuvent pas participer à cette étude. Si un participant se trouve dans une situation où il ne peut pas comprendre la situation, les conséquences de la décision de participer et participer activement, il ne peut pas donner un consentement éclairé. Si vous avez un doute sur la capacité d'un participant de consentir, ne continuez pas avec la procédure. Contactez votre superviseur pour la suite de la procédure.

Quand le participant a droit à participer dans l'étude et est capable de consentir, vous commencez le processus de consentement éclairé. Commencez par donner des informations générales sur l'étude, ses objectives, le déroulement et le temps que la participation va prendre. Permettez aux participants de poser toutes les questions qu'ils ont. Il y a deux procédures de consentement éclairé dépendant si le participant est lettré ou illettré:

Personne lettrée
Donnez le formulaire de consentement au participant. Donnez-lui le temps nécessaire pour lire et comprendre et pour poser toutes les questions qu'il a. Répondez à toutes les questions posées et après, donnez le temps nécessaire pour prendre une décision. Lorsqu'il est d'accord, vous écrivez le nom du participant, la date et il signe. Vous signez le formulaire. Une copie est remise au participant et une copie est gardée dans son dossier.

Personne illettrée
Si le participant est illettré, il vous faut un témoin impartial pour la procédure de consentement éclairé. Donnez un exemplaire du formulaire de consentement éclairé au témoin. Expliquez le consentement dans la langue que comprennent le participant et le témoin. Le témoin s'assure que les explications données sont conformes à ce qui est écrit. Répondez à toutes les questions posées. Assurez-vous que le participant n'a plus de question et donnez-lui le temps pour prendre une décision. S´il est d'accord, vous écrivez le nom du participant et la date. Le participant met une croix sur le formulaire, le témoin met son nom, date et il signe. Vous écrivez votre nom, la date et signez. Une copie est remise au participant et vous gardez une copie dans votre dossier. Donnez assez de temps pour le participant de décider s'il veut participer à l'étude ou non.

5. <u>COLLECTE DE DONNÉES QUALITATIVES</u>

Dans cette partie, on présente des principes fondamentaux de collecte de données qualitatives; comme le rôle d´enquêteur, la technique d'interrogation, le contenu des questions et comment on peut encourager les participants de raconter plus.

5.1. Règles générales pour la collecte de données qualitatives

Il y a plusieurs règles et des "bonnes pratiques" pour des collecteurs de données qui doivent être respectés pendant la collecte de données qualitatives. Ces règles vont assurer la qualité des données. Ci-dessus, on explique toutes ces pratiques. Veuillez lire soigneusement et poser des questions s'il y a des doutes. Veuillez suivre ces pratiques pour tous les entretiens que vous ferez.

Règle générale 1 : Garantissez un cadre confidentiel

Il est important de protéger la vie privée des participants. Pour obtenir des bonnes et honnêtes réponses, il est important que le répondant se sente à l'aise, de faire l'enquête en confidentialité et de se rassurer que personne d'autre ne peut entendre la conversation entre vous et le participant. Essayez de trouver un lieu où vous êtes seul avec le participant. Si nécessaire, demandez aux membres de la famille de sortir de la chambre ou de s'éloigner.

Règle Générale 2 : Restez neutre

Il est important que vous adoptiez une attitude neutre à l'égard des participants et que vous ne leur jugez pas. Il y a 2 raisons pour maintenir une attitude neutre : 1) pour obtenir des réponses qui ne sont

pas influencées par vous ; 2) pour assurer que les participants n'ont pas honte, ne se sentent pas gênés et pour maintenir des bons rapports avec eux. Cela est essentiel pour l'étude.

Dans cette étude, on demande aux participants de partager des détails de leurs vies. Gagner leur confiance et établir des bons rapports avec eux sont des components cruciaux pour le succès de cette étude. S'ils se sentent jugés par des membres de notre équipe, qui réalisent l'étude afin d'améliorer les services de santé, il peut arriver qu'ils ne souhaitent pas participer à l'étude. Cela peut compromettre la collecte de données, et l'étude dans son ensemble. Soyez prudent que rien dans votre choix de mots ou d'attitude ne cause pas un criticisme, surprise, jugement, approbation ou désapprobation des questions posées ou des réponses données par le participant. Un participant pourrait vous raconter quelque chose que vous trouvez difficile à croire ou extrême. Efforcez-vous de maintenir des expressions faciales, un langage du corps et des réponses neutres. L'équipe de recherche ne va pas tolérer n'importe quelle forme de manque de respect envers les participants.

Pour gagner la confiance des participants et les mettre à l'aise, soyez détendu et naturel. Soyez amicale et ouvert et présentez-vous. La manière de vous présenter, un langage corporel agréable et le fait de ne pas utiliser un langage stigmatisant/jugeant vont aider à mettre les participants à l'aise et, enfin, au succès de l'étude. Le questionnaire utilisé pendant cette étude est formulé pour être neutre et pour ne pas contraindre le participant à répondre d'une certaine façon. Il est aussi dessiné pour ne pas stigmatiser des participants.

Si un participant vous donne une réponse ambiguë, ne devinez pas ce qu'il a voulu dire. Si vous dites quelque chose comme « Oh, oui, je pense que vous voulez dire… » il est très probable qu'ils vont être d'accord avec vous quoi que vous disiez. Si un participant donne une réponse ambiguë, demandez-lui d'éclaircir sans faire une assomption sur ce qu'il a voulu dire.

Règle Générale 3 : Adoptez l´attitude de la personne extérieure naïve

On ne peut qu'analyser ce que les participants ont dit. Quelques fois les participants vont vous dire quelque chose dans une manière courte parce qu´ils pensent que vous savez déjà ce qu´ils veulent dire. N´êtes pas contents avec cela. Peut-être que vous avez déjà entendu cela plusieurs fois. Mais s´il n´y a pas une réponse complète enregistrée, on a des difficultés pour l´analyser. Ce qui n´est pas enregistré, le participant n'a pas dit. Adoptez une attitude naïve pour que le participant explique toute l´histoire. Prétendez de ne pas comprendre sans explication pour que le participant donne l´image complète.

Règle Générale 4 : Faites les participants répondre avec une profondeur suffisante

Quelques fois les participants donnent des réponses courtes. Il y a peu de questions que cela suffit. Le but d´un entretien qualitatif est de comprendre les points des vues, les attitudes et les motivations des participants. Cela ne fonctionne que si les participants ont la possibilité de raconter beaucoup et de répondre avec une profondeur suffisante aux questions. Vous pouvez encourager le participant à expliquer sa réponse, à clarifier ce qu´il veut dire ou à donner un exemple. Dans les guides du sujet, vous trouvez souvent les mots « Questions exploratoires » et une instruction ajoutée. C´est une aide pour recevoir une réponse plus longue ou pour guider le participant à penser à un certain aspect de cette question. Quand le participant a déjà donné une réponse qui couvre cet aspect on peut sauter l´instruction. Dans l´autre cas, on mentionne cet aspect et attend la réaction du participant. La qualité des entretiens qualitatifs est dépendante de la profondeur des réponses des participants!

Règle Générale 5 : Ne montrez pas les questions aux participants

Les participants peuvent être influence dans leurs réponses s'ils voient ce qui vient après. Ne montrez pas les questions aux participants.

Règle Générale 6 : La liberté de l´enquêteur

Comme enquêteur, vous avez plus de liberté dans les entretiens qualitatifs que dans les entretiens quantitatifs. Vous êtes libre de rester plus longtemps avec une certaine question quand vous avez l´impression que c´est un sujet important pour les participants. Le but d´un entretien qualitatif est de comprendre ce qui est important pour les participants concernant ce sujet en général. Si vous n´avez pas assez de temps, vous avez aussi la liberté de sauter une question. On parlera en avance des questions les plus importantes qui doivent être demandés en tout cas, et en profondeur. ***Règle Générale 7 : Écoutez bien***

a) Bonne écoute
- Utilisez le langage corporel pour montrer aux gens que vous écoutez et comprenez
- Utilisez l'expression du visage pour montrer votre intérêt et réfléchissez sur ce qui se dit
- Écoutez la façon dont les choses sont dites à travers le ton et le langage corporel de quelqu'un
- Posez des questions pour clarifier les problèmes
- Résumez et reformulez les problèmes afin de vérifier la compréhension de ce qui a été dit et demande un retour d'information

b) Mauvaise écoute
- L'enquêteur s'est détourné de la personne qui parle
- Il a les bras croisés
- Il ne regarde pas la personne en question dans les yeux
- Il ne pose pas de questions de suivi
- Il pose des questions sur des choses que l'orateur a déjà évoquées (ne pas montrer l'écoute active)

Règle Générale 8 : Pratiquez débriefing fréquemment

Le débriefing est une partie essentielle de la collecte de données qualitatives qui améliore la qualité des résultats. Le coordinateur de recherche rassemble l'équipe de recherche pour une réflexion immédiate sur les expériences de ce même jour.

Le coordinateur de la recherche devrait prendre des notes sur les commentaires des interviewers et des preneurs de notes. Ces notes sont aussi des données! Elles peuvent grandement influencer l'analyse des données et identifier les difficultés qui peuvent être résolues à temps en modifiant les questionnaires.

Sans débriefing, s'il y a un problème dans l'un des entretiens, tous les entretiens suivants seront affectés de la même manière. Avec le débriefing, nous pouvons identifier les problèmes susceptibles de rendre les données inutilisables et les corriger lors des entretiens postérieurs.

Le débriefing est également important car il nous permet de réfléchir à différents points de vue. Un autre enquêteur peut remarquer quelque chose de significatif que nous n'aurions pas autrement. Assurez-vous d'allouer le temps approprié à ce processus. Il est normal d'être fatigué à la fin de la

journée, mais le débriefing est aussi un moyen de renforcer l'équipe, d'apprendre ensemble et de se soutenir et encourager mutuellement.

Choix du moment:
• Retour en voiture au bureau • À la fin de la journée de travail

Questions clés:
• Quels sont les thèmes communs abordés par les participants?
• Qu'est-ce qui a fonctionné pour chaque outil et qu'est-ce qui n'a pas fonctionné?
• Qu'est-ce qui a fonctionné en générale? Qu'est-ce qui n'a pas fonctionné? Quels changements devraient être faits?

Suivez la discussion:
• Enregistrez la discussion
• Suivez les modifications apportées aux outils
• Suivez la justification des modifications apportées aux outils

5.2. Evènements défavorables

Les évènements défavorables sont des choses négatives qui peuvent arriver au cours de l'étude. Si un évènement défavorable arrive à un des participants qui est lié à l'étude ou arrive pendant la mise en œuvre de l'étude (p.ex. mort, violence, harcèlement, troubles mentaux/psychologiques), appelez votre superviseur ou le responsable de la recherche.

6. L'UTILISATION DU DICTAPHONE

L'enregistrement est l'étalon-or en matière de collecte de données dans des entretiens qualitatifs. Il améliore la qualité des données, facilite le travail des enquêtrices et des preneurs de notes et permet la transcription et analyse des données. L'intention d'enregistrer devrait être clairement énoncée dans l'introduction et dans le formulaire de consentement éclairé. Le participant doit comprendre l'intention d'enregistrer et doit accepter et donner son autorisation avant.

Avant d'enregistrer, assurez-vous de couvrir les points suivants:
• Recevez le consentement de tous les participants
• Expliquez pourquoi vous enregistrez la discussion ou l'entretien
• Insistez sur l'importance d'une information précise
• Expliquez que la confidentialité sera protégée
• Invitez des questions

Comment fonctionne le dictaphone?

1) Allumer le dictaphone : Déplacez le bouton le plus bas à la côté droite.

2) Éteindre le dictaphone : Pressez le bouton « play/pause » pour quelques secondes. L'appareil vous montre « bye-bye » et quitte le mode de travail. Pour l´éteindre complètement vous déplacez le bouton le plus bas à la côté droite. C´est important de le faire dans cet ordre pour ne pas endommager les données!

3) Commencer l´enregistrement : Pressez le bouton « rec » courtement. Quand la lumière d´enregistrement montre une lumière constante et le temps en haut commence à changer, vous êtes en train d’enregistrer.

4) Pauser l´enregistrement : Pressez le bouton « rec » courtement. La lumière d´enregistrement clignote et le temps cesse de changer. Pressez le bouton « rec » encore une fois pour continuer l´enregistrement.

5) Sauver l´enregistrement : Pressez le bouton « play/pause » pour quelques secondes. L’appareil vous montre « saving » et « bye-bye » et quitte le mode de travail.

6) Connecter le dictaphone avec l´ordinateur : Utilisez le cable USB et connectez-le avec le dictaphone et l´ordinateur. Vous pouvez utiliser le dictaphone comme un USB et télécharger les données sur l´ordinateur. Retirez-le dictaphone proprement pour ne pas endommager les données!

7) Charger le dictaphone : Connectez le dictaphone et votre ordinateur avec le cable USB. Pour un chargement complet, cela durera 4 heures. Il vous montrera une batterie complètement chargée qui ne clignote plus. Laissez-le dictaphone allumé pendant le chargement.

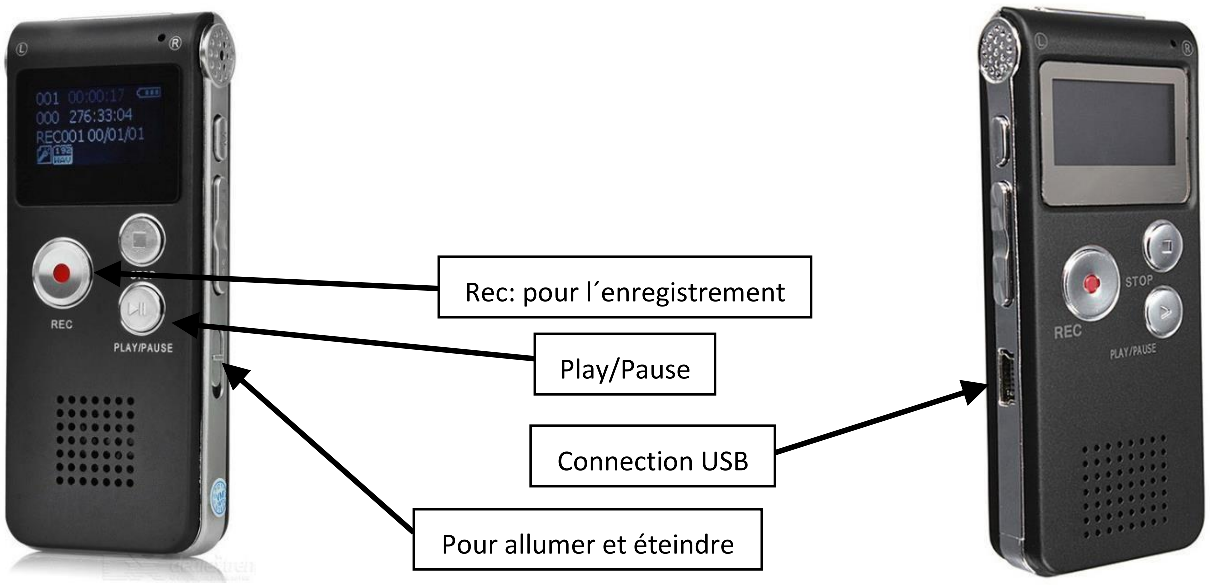

Quelques points logistiques à considérer:

- Vérifiez la batterie avant de partir pour visiter l’école
- Placez l’appareil d’enregistrement dans un emplacement central afin que les voix de tous les participants soient bien entendues
- Assurez-vous de laisser l'appareil d'enregistrement allumé même après la fin de la partie formelle de l'entretien: les meilleures informations peuvent venir à la fin quand les participants sont plus détendus!
- Une fois l'entretien terminé, téléchargez l'enregistrement sur l’ordinateur, puis effacez le fichier dans le dictaphone.

ANNEXE : Exemple de modèle socio-écologique

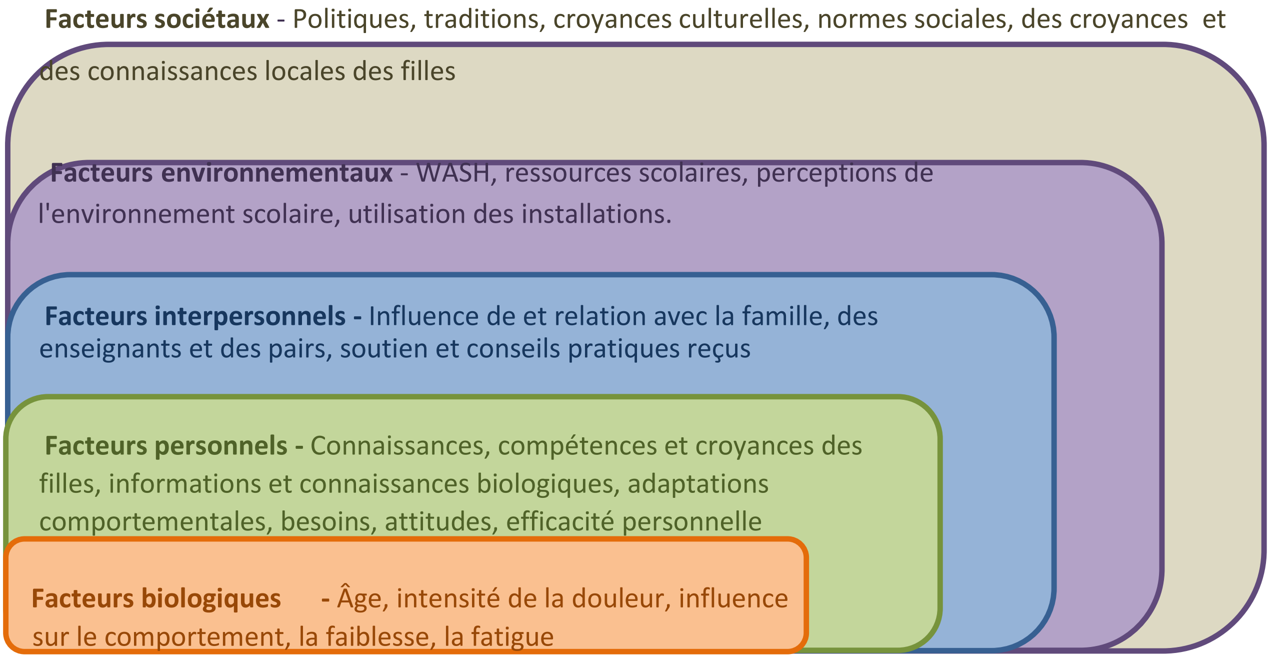

WASH – L'Eau, L'Assainissement et l'Hygiène. Image adaptée et traduite du WinS4Girls E-Course de l'UNICEF et l'University Emory

Appendix 2b: Written manual for data collectors (English version)

Index

1. INTRODUCTION 25
   1.1. Context of the study 8
   1.2. Objectives of the study 8

2. THE STUDY.. 27
   2.1. Study site 27
   2.2. Study design 27

   2.3. Organisation of the survey 6
   2.4. Study tools 7
   2.5. Focus group discussion 8
   2.6. In-depth interview 9
   2.7. Your role as an interviewers 9
   2.8. Supervision of the study 10

3. SANITARY PRODUCTS 10

4. GENERAL PROCEDURES 11
   4.1. Inclusion and exclusion criteria 12
   4.2. Informed consent 12
   Literate participants 13
   Non-literate participants 13

5. QUALITATIVE DATA COLLECTION 13

4.1. General rules for qualitative data collection 13
4.2. Adverse events 16

6. USE OF THE DIGITAL VOICE RECORDER 16

1. INTRODUCTION

1.1. Study site

The onset of menstruation can be a difficult time for many girls around the world and can be a challenge to their physical, mental and social development and well-being. Many times they do not know what is happening to them or see it as an illness. They often feel alone and have no one to talk to. The subject is taboo and many social rules can lead to their isolation. They may also suffer from other health problems that result from improper menstrual hygiene. In other countries, studies have shown that girls may also resort to unsafe practices to obtain sanitary products, which are often difficult to access.

Girls frequently begin to miss school or drop out altogether. Some of the reasons given are lack of facilities at school, teasing by classmates, difficulty concentrating in school, self-esteem issues, etc. For these and other reasons, menstrual hygiene management has received increasing attention in recent years, particularly as the health and education sectors seek to reduce education disparities, promote gender equality and empower women and girls.

We would like to understand what the reasons are from different angles so that the institutions involved can develop or improve programs that will help them. Each country and region is very different, as are the people who live there, and there are often many socio-cultural beliefs and regional practices that have great influence, that we would like to examine. All these aspects will be explored during the interviews.

1.2. Study objectives

This work seeks to understand the current situation with regard to menstruation and to identify the challenges and facilitators of menstrual hygiene management in Nouna (Burkina Faso), whether

economic, socio-cultural and/or infrastructural. This formative research phase will also test the feasibility and acceptability of various sanitary products on a very small scale. The results of this research will form a second phase of research in 2019/20, which will examine the outcomes associated with delivering menstrual hygiene management products on a larger scale.

2. THE STUDY

2.1. Study site

This study will be conducted in the Nouna Health and Demographic Surveillance Site (HDSS), in the province of Kossi. It will take place at the Nouna Health Research Centre (NHRC). Much of this area is rural and 30% of the population lives in the town of Nouna. According to the most recent data (2015), approximately 107,000 people lived in the HDSS census area. According to a recent “Adolescent Health” study from the Heidelberg Institute of Global Health (HIGH) on female genital mutilation at the Nouna HDSS, girls between the ages of 12 and 20 suffer from a severe lack of knowledge about menstruation. More than 80% of rural women in Burkina Faso use old cloths to manage their periods.

2.2. Study design

The study was developed in cooperation between NHRC and the HIGH at the University of Heidelberg. There will be two phases of this study.

Work package	Type of qualitative data collectes	Source of the data
1. Knowledge generation phase: current attitudes, experiences and practices related to menstruation and menstrual hygiene management	• Observations<br>• Focus group discussions<br>• Semi-structured indepth interviews	School observations<br>Focus group discussions with schoolgirls and mothers (divided by respondent group)<br>In-depth interviews with teachers and key informants (representatives involved in policies or interventions in water and hygiene in schools, from *UNICEF*, nongovernmental organisations, Ministry of Health or Education and/or school board members)
2. *Mini-testing* phase: feasibility and acceptability of the products	• Semi-structured indepth interviews<br>• Diary study	In-depth interviews with schoolgirls pre and postdistribution of the products<br>Schoolgirls’ diaries

c. General perspectives phase

Observations: We have already collected information on secondary schools in Nouna, including the number of female teachers, the number of girls who are over 12, access to water, latrines, the presence of places to change, etc. This is an important step in assessing whether distributing a product to schoolgirls is feasible (on a small scale or on a larger scale for a later phase of this work).

Focus group discussions (FGDs): Up to 15 FGDs with 6-8 participants per group will be held, including schoolgirls and mothers. Questions will focus on beliefs in relation to menstruation, their personal experience, knowledge about menstrual hygiene management products, main barriers to accessing products, local perception of products and the feasibility and acceptability of potential products. Furthermore, we aim to explore societal factors, such as taboos or opinions regarding menstruation

and sanitary products. Please read the questionnaires carefully before the corresponding day of your training.

In-depth interviews (IDIs): Semi-structured interviews, including up to 10 teachers and up to 15 key informants (such as representatives from UNICEF, non-governmental organizations, Ministry of Health or Education and/or school board members) will be conducted. They will include questions that focus on existing standards and how different menstrual hygiene management interventions could be integrated into existing structures. We will try to understand: How is hygiene and particularly feminine hygiene generally in schools? Who might be best placed to guide school girls in terms of menstruation and menstrual hygiene? How should this conversation take place and what should the conversation include or exclude? What is most achievable in terms of improving menstrual hygiene management in schools, whether in terms of product type (pads versus menstrual cups or padded underwear) or product distribution? Please read the questionnaires carefully before the corresponding day of your training.

Pre-distribution IDIs with schoolgirls: The selected group of schoolgirls from the FGDs will participate in semi-structured IDIs before receiving the sanitary products. These interviews will examine their general perspectives on menstruation and their experience and knowledge. There are also questions about menstrual hygiene management (at school, at home, sanitary products they know, etc.) and about their recommendations and needs. These interviews will provide a more private environment where the girls can share more about the themes discussed during the group discussions.

d. ***Mini-testing*** phase

We will present the products in group discussions with school girls and mothers. A selected group of schoolgirls will receive sanitary products which will be tested for a period of two months. The alternatives will be presented in this manual in the chapter "2.7. Sanitary products". Each participant will be able to choose and try a single product. Up to 30 schoolgirls will be selected for this phase.

Post-distribution IDIs with schoolgirls: After 2 months of using the products, another semi-structured IDIs will be devoted to the details of their experience with the sanitary products. During these interviews, we want to understand the girls' opinions on different aspects of the distribution process, the knowledge they would like to acquire, the type of products they prefer, the difficulties they face in using them and the support received (Who would they like to talk to? What questions did they have? Who should give them the products? etc.). This will be an interview in which we ask them to design their ideal program for menstrual hygiene management, detailing the changes in the way we have done it. We will further explore their experience with the diary and discuss the issues within it.

Diary: Participants will receive a diary containing questions about their experience with the product, which will be discussed during the post-distribution interview. There are some ideas or thoughts that they might forget during the 2 months of use. Time changes the quality and intensity of our memories. Things that seemed good or bad at first may seem different after a while, when we get used to them. The journal allows them to write down their thoughts on the products as they discover them. Please read the questionnaires and the diary carefully before the corresponding day of your training. The following table is a summary of the sample size based on these described participant groups.

Type of interview	Quantity of interviews	Number of participants	Maximum sample size
FGDs with mothers	7-8 groups	6-8 mothers/group	
FGDs with schoolgirls	7 groups	6-8 girls/group	

IDIs with teachers	≤10 interviews	≤10 teachers	64
IDIs with key informants	≤15 interviews	≤ 15 key informants	56<br>10
Pre-distribution IDIs with schoolgirls	≤30 interviews	≤30 schoolgirls	15<br>30
Post-distribution IDIs with schoolgirls	≤30 interviews	≤ 30 schoolgirls	30

These are the numbers established in agreement with the Ethics Committee of Nouna and Heidelberg. Therefore, these limits must be respected. On the other hand, the number of participants can be lower, if justified.

2.3. Organisation of the survey

The actual order of IDIs and FGDs will be a little different due to practical and logistical issues.

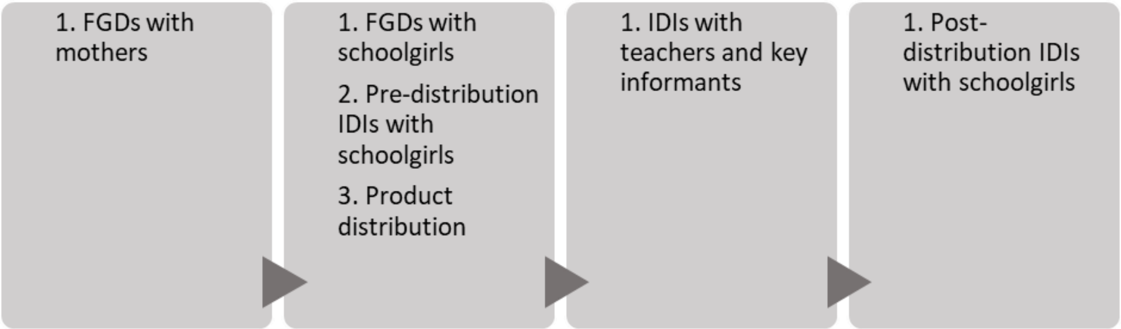

During the first week, we would have FGDs with the mothers. This will allow us to have consent forms signed, in the case of girls under 18 years old. It will also save us time, as we will only need to bring the mothers together once.

Once these FGDs are complete, we will conduct them with the girls. Usually 1 FGD group per day followed by 2-3 IDIs with the selected girls. It depends on the number of interviewers available. It is important that FGDs and IDIs take place on the same day, because we want the choice of product to be as neutral as possible (that the student decides for herself). Immediately after the pre-distribution interview, we will give them the chosen sanitary product.

Once all FGDs are complete, we can continue IDIs with teachers and key informants. Finally, 2 months after giving the sanitary products to the girls, we will carry out post-distribution IDIs.

The project schedule may vary depending on many factors. Our goal is not to do a lot of interviews a day to finish quickly. Investigators should be aware of their skills and limitations and plan accordingly. It is normal to be tired after several interviews and our ability to lead the conversation, actively listen and recognize the points worth exploring is a very exhausting process. This is why doing too many interviews in one day can result in poor quality data.

The following timeline is simply an estimate of how long we think each of the phases will take:

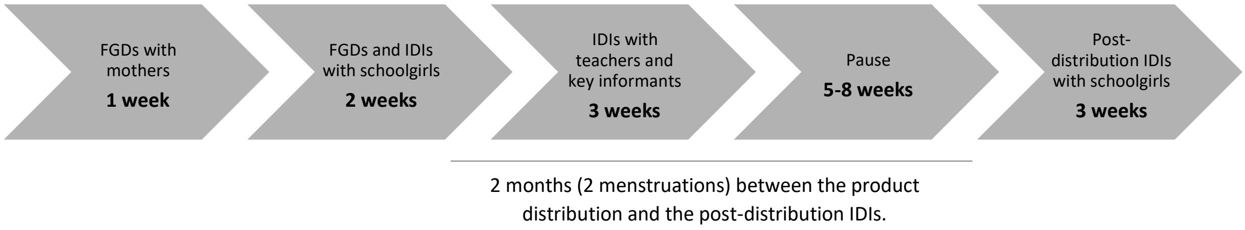

2.4. Study tools

The study tools will be adapted during training and piloting, and can then be adapted again according to the results of the first FGDs and IDIs. Questions are never definite, we can always make changes, if needed. The modification of the questionnaires is essential for the quality of the qualitative data. The points highlighted next are also just some of the topics to explore. During the FGDs and IDIs, other topics raised by the participants might become significant for the study and you are encouraged to pursue them.

a. FGDs with mothers

Objective: To investigate the role of mothers in communicating knowledge, information, attitudes and practices regarding menstruation to their daughters; To understand mothers' views on the challenges girls face in schools.

Areas of inquiry:

(1) Women's knowledge, perceptions and behaviors;

(2) Mothers' support for daughters and their understanding of their experiences; (3) Mothers' understanding of girls' experiences in school;

(4) Recommendations.

b. FGDs with schoolgirls

Objective: To study and identidy the experiences, challenges and needs of a typical girl.

Areas of inquiry:

(1) Characteristics of actual and ideal school infrastructures;

(2) Life changes and social norms;

(3) Experiences and challenges in relation to menstruation; (4) Recommendations to improve girls’ experiences.

c. Pre-distribution IDIs with schoolgirls

Objective: The interview topics are similar, but they could share more privately. We also talk more about specific sanitary products and their needs.

Areas of inquiry:

(1) Knowledge and perceptions about menstruation and menstrual hygiene management;

(2) Personal experience with menstruation; (3) Behaviours during menstruation;

(4) Recommendations.

d. Post-distribution IDIs with schoolgirls

Objective: Discover the best way to distribute a product and practical steps to improve their experience.

Areas of inquiry:

(1) Their experience with the chosen sanitary product;
(2) Their experience with the diary; (3) Creation of a hypothetical ideal program; (4) Recommendations.

e. IDIs with teachers

Objective: To obtain information about the school and teachers' views on problems related to menstruation in the school environment.

Areas of inquiry:

(1) Social norms and behaviours;
(2) Teachers perceptions on menstruation and menstrual hygiene; (3) Support systems that could be developed;
(4) Recommendations.

f. IDIs with key informants

Objective: To identify existing systems to strengthen and good practices.

Areas of inquiry:

(1) Perceptions of the population, institutions and government;
(2) Challenges linked to menstruation and menstrual hygiene and their professional experience; (3) Programs and campaigns already implemented;
(4) Recommendations.

2.5. Focus group discussions

For a FGD, we bring together a group of people who meet the inclusion criteria. The group has a moderator and another person taking notes. The discussion is recorded and transcribed. During the FGD, we want to listen to the participants and their general ideas on the subject concerned. We want to understand the attitudes and experiences of the participants. We also gain information about the natural environment of the participants because they are in a group and we can observe the interactions. Sometimes, participants are asked to complete an exercise, such as writing words on a board or discussing pictures.

The moderator is the person who guides the discussion. He tries to put the participants at ease and signals to them that we listen to what they say. There is an interview guide with questions that the moderator asks. It is very important that the participants are encouraged to share and for their answers to have a certain depth. There are a few strategies to receive sufficient responses: one can probe by asking for an explanation, clarification, specification, example or personal opinion. The rules for qualitative interviews in general are explained later in this manual.

The person taking the notes is the group's observer. In a focus group, it is not only the responses of the participants that are important but also their interactions. How do others react when a participant explains her opinion? Do they agree? Is there a hierarchy in the group? The observer can also note

when there are problems understanding certain questions, when he feels that the participants are tired or other things that could be important to notify. The observer helps the moderator with the introduction to participants and ensures that the recording works well.

2.6. In-depth interviews

The purpose of a qualitative interview is to gain deep insights into the participant's viewpoints and to understand their interpretations of the world. We have an interview guide, but we can formulate the questions differently, if the participant does not understand them. The interview guide ensures that the interviewer covers the same topics with each participant. It is important that the participant is encouraged to share more and that his answers are not too short or superficial. The interviewer can ask him to specify, explain or give an example when he feels that the answer is not detailed enough. The interview is recorded and transcribed afterwards. The interviewer tries to behave in a way that the participant feels comfortable and that he can be trusted. This is called good reporting. In the best case, the participant has the impression of having a conversation rather than an interview. Some strategies for developing rapport include: maintaining eye contact, nodding to encourage the participant, commenting on the response when appropriate and not interrupting the participant.

2.7. Your role as an investigator

As an investigator you are responsible for data collection. You will seek consent and conduct FGDs with women and girls and IDIs with girls, teachers and key informants. Your cooperation is of the utmost importance, serves to guarantee the quality of the data collected and helps us to meet the study schedule.

Collecting high quality data is very important for a successful study. In the future, the data can be used to improve health services in the region and the living situation of women and girls. As an investigator, you are part of a research team. In the team, it is important that we identify problems that arise during the implementation of the survey and solve problems related to the data collection so that they do not accumulate and make the data unusable. You will record FGDs and IDIs. When collecting data, it is important that you respect the participants, listen carefully and ask the questions in a way that the participants answer with sufficient depth.

If you are the note taker/observer, your role is also indispensable. Please remember that ratings are data too! They are taken even when a digital voice recorder is used. You should note the main issues raised, body language and non-verbal communication, group interactions, spoken/colloquial language, interruptions, people in the group who dominate the conversation and people who are generally quiet. These notes are essential for debriefing, transcription and data analysis. Please make sure you have these skills in advance.

In any case, interviewer or note taker, your work will be checked regularly by a research coordinator. Good results can be obtained only by conscientious work. You can always chat with or ask for help from one of the coordinators if you have any problems or concerns whatsoever. We hope you will learn and gain valuable research skills during this study. Remember that learning is an ongoing process and hard work brings the best personal and scientific results.

2.8. Supervision of the study

This study is a cooperation between NHRC, in Burkina Faso, and the HIGH, in Germany.

Dr. McMahon, the Principal Investigator, has been conducting research in the areas of maternal, child and related health issues in many sub-Saharan African countries for 9 years. She has conducted studies on menstrual hygiene management in Kenya and on the health and empowerment of school girls in Mali. As head of the Behavioural Interventions Group at the HIGH, she has spent the past year working closely with NHRC to develop health interventions related to pregnancy and nutrition.

Dr. McMahon will work closely with Burkinabe researcher Dr. Ali Sié and Dr. Sié's colleague, who oversees qualitative research, Ms. N. Hélène Sawadogo. Dr. Sié has been the director of the NHRC since 2007 and has worked at the NHRC since 2004. He has conducted several scientific surveys on health conditions and health systems. Ms. Sawadogo is the co-lead for qualitative research on nutrition and health, which has led to a strong partnership between her and Dr. McMahon.

3. SANITARY PRODUCTS

In this part of the manual, we will present the products that will be offered to the schoolgirls after the interview. It is important that girls choose freely. Their choices are also data. In this way, we want you to remain as neutral as possible when presenting the products during group discussions. During this part of the training, you will be able to see the products and learn about them. This will be a good time to share your opinions, thoughts and expectations regarding each of the products. Please feel free to ask questions about them. All of these products have been tested and are safe to use.

ThinX period underwear

Period underwear wear have a capacity of 2 sanitary pads. They have to be washed with cold water and soap, and dried in the sun o in a dry place.

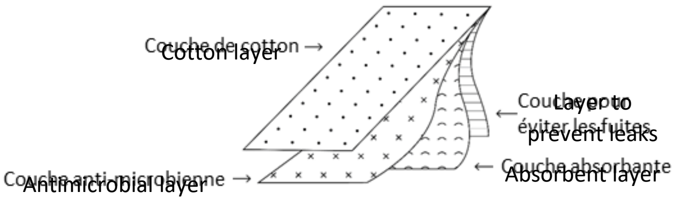

Palobdé reusable sanitary pads

These sanitary pads are made of cotton. Each sanitary pad comes with an impermeable layer. Reusable pads need to be changed every few hours and washed with soap after use. They need to be dried in a dry place. They can be reused for two years. They are made in Burkina Faso.

Disposable sanitary pads

They need to be regularly changed as well, depending on menstrual flow. They should not be thrown on the ground, or in toilets, after use. They need to be burnt or thrown in special trash cans.

Menstrual cup

The menstrual cup needs to be put in boiling water during 3-5 minutes at the beginning and at the end of every menstruation, before returning it to its bag. You need to wash your hands with water and soap before introducing it and before changing it. The cup needs to be washed as well, before reintroducing it. You should empty it every 12 hours approximately. If the cup falls in the toliets or on the ground, do not reintroduce it. You can put it in boiling water before using it again. You can reuse it for up to 10 years.

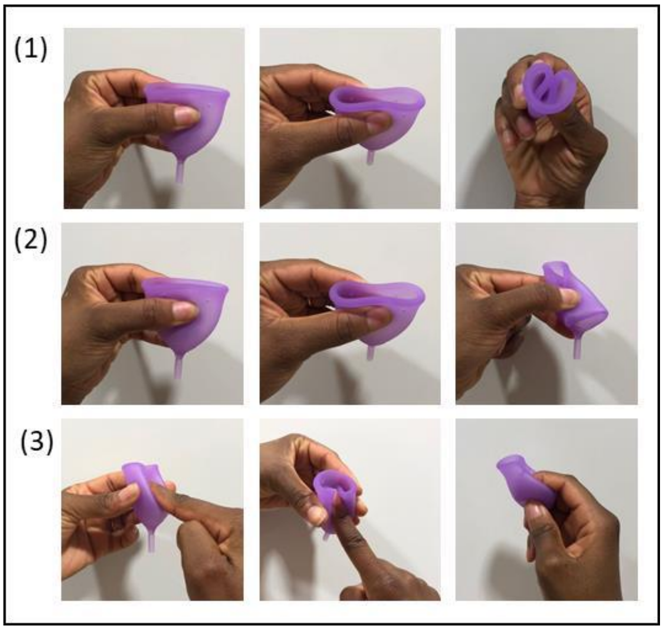

Here, you can see the three methods to fold it to introduce it.

4. GENERAL PROCEDURES

4.1. Inclusion and exclusion criteria

When you are in the field and you have found the participant with the list of participants, you will consider again, if the participant meets the inclusion criteria. Ethnicity, race, political orientation, religion and class are not criteria for inclusion or exclusion in this study.

To be able to determine the ultimate objective of the subsequent trial (which will assess the impact of the provision of sanitary products on school attendance), it is necessary to include girls from the time of menarche (from the age of 12). Since we aim to determine a feasible and acceptable solution in the field of menstrual hygiene management for women in the context of marginalized areas, we will be able to provide this solution to women from birth.

Engaging younger girls in education about menstrual hygiene and safe menstrual practices through the provision of sanitary products is one way to address the challenges and barriers associated with this age group, which are likely to differ from people related to older women.

Inclusion criteria	Exclusion criteria
Age 12 or more<br>Able to give informed consent, if > 17 years old<br>Willing and capable of giving informed consent and with a parent/tutor willing and capable to give informed consent, if < 17 years old	Age less than 12<br>Incapable of giving informed consent<br>Parent/tutor refuses to give informed consent, if <17 years old<br>Participant refuses to provide informed consent

4.2. Informed consent

The informed consent is a voluntary consent to participate in research. Consent is a dynamic process, not just a signature on a paper. It continues throughout the study and it is never final! In order to

obtain informed consent, it is essential that the participant fully understands the information given on the research and the risks and benefits of participating in the study. The goal is that the individual receives all the information necessary to reach an informed decision on whether or not to participate in the study, without being subjected to pressure, influence or intimidation. In the case of schoolgirls under the age of 18, you will need to obtain informed consent from their parents or guardians and follow the same informative process with both, the girl and her guardian/parent.

The informed consent process is a dialogue about the objectives of the study, its duration, procedures, alternatives, risks and benefits. This is a process that continues throughout the study: participants can withdraw consent at any point in the study. It is your responsibility to ensure participants understand all the information, why they are joining the study and what giving an informed consent means.

The informed consent form is an important part of the informed consent procedure. This sheet includes information on the purpose of the research, the investigation procedures, the risks, potential benefits of this research for society and individuals, the time the investigation will take, contact information for the principal investigators, a declaration that participation in the study is voluntary, the right to confidentiality and the right to withdraw from the study at any point without consequences.

In this study, the informed consent form is a paper form. It will contain the participant's identifying information, including their signature or a fingerprint, if they are illiterate. It is extremely important that you save this document and that its contents are not seen or read by other people.
Sometimes, an individual cannot give informed consent. In this case, they cannot participate in this study. If a participant finds himself in a situation where he cannot understand the situation, the consequences of the decision to participate and actively participate, he cannot give informed consent. If you have any doubts about a participant's capability to consent, do not continue with the procedure. Contact your supervisor for the rest of the procedure.

When the participant is eligible to participate in the study and is able to consent, you begin the informed consent process. Start by giving general information about the study, its objectives, the procedure and the time that participation will take. Allow participants to ask any questions they have. There are two informed consent procedures depending on whether the participant is literate or illiterate:

Literate person

Give the informed consent form to the participant. Give him the necessary time to read and understand all the information presented. Answer all the questions he might have and give the necessary time to decide afterwards. Once he has decided, write the name of the participant, the date and ask him to sign. You will also sign the form yourself. A copy is given to the participant and a copy is saved in his file.

Illiterate person

If the participant is illiterate, you need an impartial witness for the informed consent procedure. Give a copy of the informed consent form to the witness. Explain the consent in the language understood by the participant and the witness. The witness ensures that the explanations given are consistent with what is written. Answer all the questions asked. Make sure the participant has no more questions and give him time to make a decision. If he agrees, write the name of the participant and the date. The participant puts a cross on the form, the witness puts his name, date and signature too. Write your

name, date and signature. Give a copy to the participant and save a copy in your folder. Give enough time for the participant to decide if he wants to participate in the study or not.

5. QUALITATIVE DATA COLLECTION

In this section, we present the basic principles of qualitative data collection; such as the role as an interviewer, the interviewing technique, the content of the questionnaire and how to encourage participants to tell us more.

5.1. General rules for qualitative data collection

There are several rules and “good practices” for data collectors that should be followed when collecting qualitative data. These rules will ensure the quality of the data. Below, we explain all these practices. Please read carefully and ask questions, if in doubt. Please follow these practices for all interviews you do.

General rule 1: Ensure a confidential environment

It is important to protect the privacy of participants. To get good and honest answers, it is important that the respondent feels comfortable to do the survey in confidentiality and to be reassured that no one else can overhear the conversation between you and the respondent. Try to find a place where you are alone with the participant. If necessary, ask family members to leave the room or move away.

General rule 2: Stay neutral

It is important that you adopt a neutral attitude towards the participants and that you do not judge them. There are 2 reasons to maintain a neutral attitude: 1) to get answers that are not influenced by you; 2) to ensure that the participants do not feel ashamed, do not feel embarrassed and to maintain a good relationship with them. This is essential for the study.

In this study, participants are asked to share details of their lives. Gaining their trust and establishing a good rapport with them are crucial components to the success of this study. If they feel judged by members of our team, who are carrying out the study to improve health services, they may not want to participate in the study. This can compromise data collection and the study as a whole. Be careful that nothing in your choice of words or attitude shows criticism, surprise, judgment, approval or disapproval of the questions asked or the answers given by the participant. A participant might tell you something that you find hard to believe or extreme. Strive to maintain neutral facial expressions, body language and responses. The research team will not tolerate any form of disrespect towards participants.

To gain participants' trust and put them at ease, be relaxed and natural. Be friendly and open, and introduce yourself. Introducing yourself, with pleasant body language and not using stigmatizing/judgmental language, will help the participants to feel at ease and, ultimately, the study to succeed. The questionnaire used during this study is formulated to be neutral and not to lead the participant to respond in a certain way. It is also designed so as not to stigmatize participants.

If a participant gives you an ambiguous answer, do not guess what they meant. If you say something like "Oh, yeah, I think you mean..." it's very likely that they're going to agree with whatever you say.

Instead, if a participant gives you an ambiguous answer, ask them to clarify without making assumptions about what they meant.

General Rule 3: Adopt the attitude of the naive outsider

One can only analyse what the participants said. Sometimes, participants will tell you something in a short way because they think you already know what they want to say. Do not settle for that. Maybe you have heard this many times before. If there is not a complete answer recorded, it is difficult to analyse it. What is not recorded, the participant did not say it. Adopt a naive attitude so that the participant explains the whole story. Pretend not to understand without an explanation, so that the participant gives you the full picture.

General Rule 4: Have participants respond with sufficient depth

Sometimes, participants give short answers. There are few instances when this is enough. The purpose of a qualitative interview is to understand the views, attitudes and motivations of the participants. This only works if participants have the opportunity to tell a lot and answer questions with sufficient depth. You can encourage the participants to explain their answer, clarify what they mean or give an example. In questionnaires, you often find the words “Probing Questions” and an added instruction. It is an aid to receive a longer answer or to guide the participant to think about a certain aspect of this question. When the participant has already given an answer that covers this aspect, the instruction can be skipped. If not, we can use the techniques in the instructions and wait for the participants’ reaction. The quality of qualitative interviews depends on the depth of participants' answers!

General rule 5: Do not show the questionnaire to participants

The participants may be influenced in their responses, if they know what they will be asked afterwards. Do not let participants see the questions beforehand.

General rule 6: The freedom of the interview

As an interviewer, you have more freedom in qualitative research than in quantitative. You have freedom to stay longer on a question that you feel relates to a topic that is more important to participants. The purpose of the qualitative interview is to understand what is important to participants within the one subject in general. If you do not have enough time, you still have freedom to skip questions that are not as important. We will discuss, throughout the training, which are the questions that are of higher priority, that should be asked in any case and in depth. ***General rule 7: Listen well***

a) Good listening
• Use body language to show people you are listening and understanding
• Use facial expression to show interest and reflect on what is being said
• Listen to how things are said through someone's tone and body language
• Ask questions to clarify issues
• Summarize and rephrase issues to check understanding of what was said and ask for feedback b) Bad listening

• When the interviewer turns away from the person speaking

- He has her arms crossed
- He does not look the person in the eye
- He does not ask follow-up questions
- She asks questions about things that the speaker has already mentioned (does not show active listening)

General rule 8: Practise debriefing frequently

Debriefing is an essential part of qualitative data collection that improves the quality of findings. The research coordinator brings the research team together for an immediate reflection on the experiences of that day.

The research coordinator should take notes of comments from interviewers and note takers. These notes are also data! They can greatly influence data analysis and identify difficulties that can be resolved in time by modifying the questionnaires.

Without debriefing, if there is a problem in one of the interviews, all subsequent interviews will be affected in the same way. With debriefing, we can identify issues that may render the data unusable and correct them during subsequent interviews.

The debriefing is also important because it allows us to think about different points of view. Another investigator may notice something significant that we would not have otherwise. Be sure to allocate the appropriate time to this process. It is normal to be tired at the end of the day, but debriefing is also a way to strengthen the team, to learn together and to support and encourage each other.

Choice of moment:
- In the car, on the way back to the office
- At the end of the work day

Key questions:
- What are the common themes brought up by participants?
- What has worked in using each interview tool and what has not?
- What has worked in general? What has not worked? Which changes do we need to make?

Follow the discussion:
- Record the discussion
- Track the changes made to the tools
- Document the reasons why the changes were made

5.2. Adverse events

Adverse events are negative things that can happen during the study. If an adverse event happens to one of the participants that is related to the study or happens during the implementation of the study (e.g. death, violence, harassment, mental/psychological disorders), call your supervisor or the principal investigator.

6. <u>USE OF THE DIGITAL VOICE RECORDER</u>

Recording is the gold standard for data collection in qualitative interviews. It improves the quality of data, facilitates the work of interviewers and note takers and allows the transcription and analysis of data. The intention to record should be clearly stated in the introduction and in the informed consent

form. The participant must understand the intention to record and must agree and give permission for it before.

Before recording, make sure to check the following points:

- Receive consent from all participants
- Explain why you want to record the discussion or interview
- Insist on the importance of precise information
- Explain that their confidentiality will be protected
- Invite questions

How does the digital voice recorder work?

1) <u>Turn on the recorder</u>: Move the lowest button to the right side.

2) <u>Switch off the digital voice recorder:</u> Press the “play/pause” button for a few seconds. The device shows you “bye-bye” and exits work mode. To turn it off completely, you move the lowest button on the right side. It is important to do it in this order so as not to damage the data!

3) <u>Begin the recording</u>: Press the “rec” button briefly. When the recording light shows a constant light and the time at the top begins to change, you are recording.

4) <u>Pause the recording</u>: Press the “rec” button briefly. The recording light will flicker and the time will stop. Press the “rec” button again to continue recording.

5) <u>Save the recording</u>: Press the “play/pause” button for a few seconds. The device shows you “saving” and “bye-bye” and exits the working mode.

6) <u>Connect the recorder to the computer</u>: Use the USB cable and connect it to the recorder and the computer on each matching side. You can use the recorder as a USB drive and download the data on to the computer. Unplug the recorder from the computer safely in order not to damage the data!

7) <u>Charge the recorder:</u> Connect the recorder and your computer with the USB cable. To completely charge it, you will need 4 hours. The recorder will show an icon of a full battery, that does not blink, on the upper right hand corner. Leave the recorder on while charging.

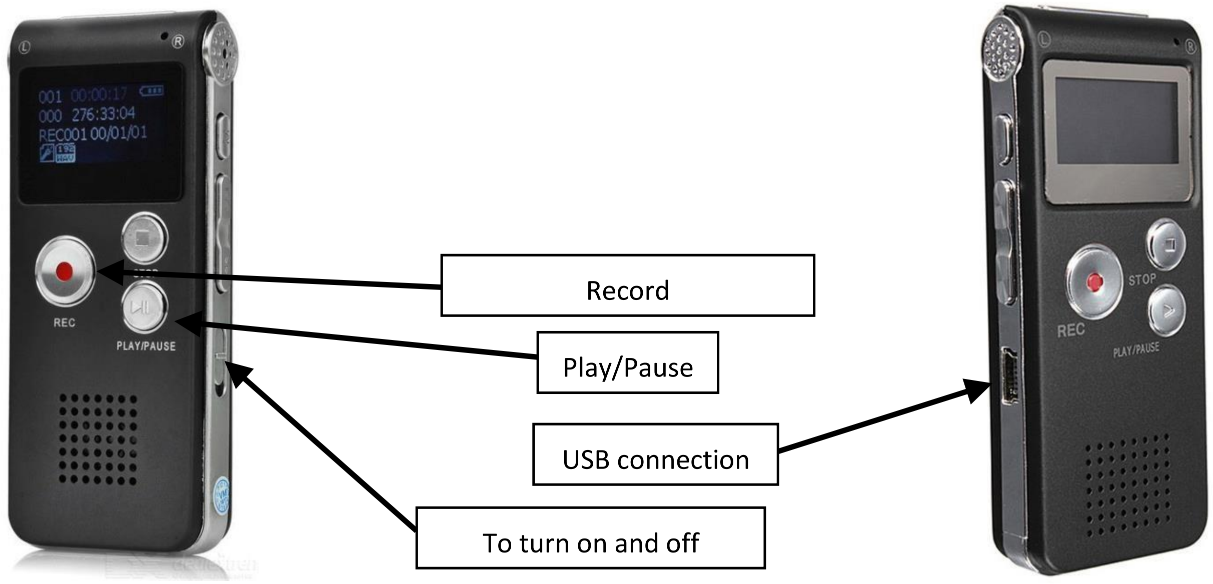

Some logistical points to consider:

• Verify the battery before leaving to visit the school.

• Place the device in a central location so that the voices of all participants are well understood.

• Ensure the device is turned on after the formal part of the interview: the best information can come when the interview has ended and the participants feel more relaxed!

• Once the interview is finished, download the recording onto the computer and erase the file from the digital recorder.

ANNEX: Example of socio-ecological model

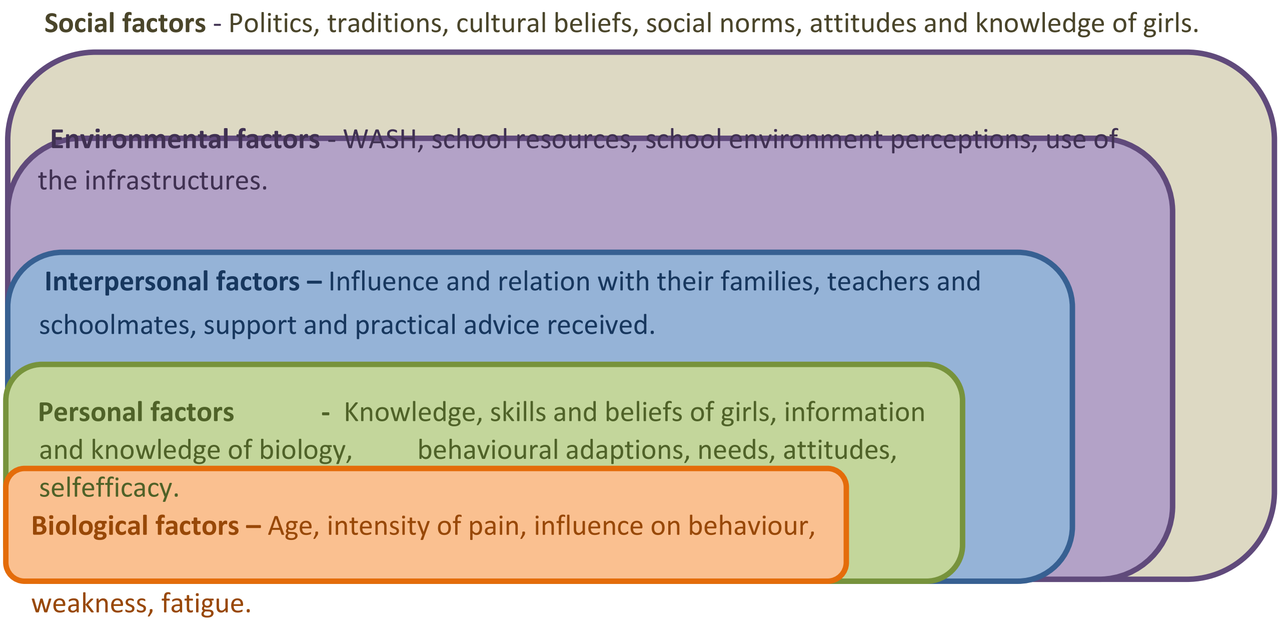

WASH – Water, Sanitation and Hygiene. Image adapted from the WinS4Girls E-Course by UNICEF and the Emory University

Appendix 3: School observation sheet

School Facility Observations[i]

A01. Research Assistant name:	A02. Date (dd/mm/yy):	A03. Start time:	A04. End time:

Basic School Information

A1. School Name:	A2. School Identification code:

B. Water observations This section will help researchers explore the water sources a school has and assess students' ability to access drinking water.

B1. Is there a water source available at school?

C. Sanitation observations This portion of the survey is meant to give researchers information about the functionality of individual toilets at the school.

C1. Are there toilets/latrines at the school?

**Toilet units**	**C1. Toilet Type**<br>**1**: Flush/ pour flush<br>**2**: Pit<br>**3**: Bucket<br>**88**: Other: _____	**C2. User**<br>**1**: Girls ONLY<br>**2**: Boys ONLY<br>**3**: Both<br>**99**: Don't know	**C3. Functionality**<br>**1**: Functional<br>**2**: Partially<br>**3**: Not Functional<br>**99**: Don't Know	**C4. Cleanliness**<br>**1**: Clean<br>**2**: Somewhat<br>**3**: Not Clean<br>**99**: Don't Know	**C5. Lighting**<br>**1**: Light<br>**2**: Somewhat<br>**3**: Dark<br>**99**: Don't Know	**C6. Locks**<br>**1**: Yes, functional<br>**2**: Not functional<br>**3.** No<br>**99**: Don't Know	**C7. Access the toilet without assistance?**<br>**1**: Yes<br>**2**: No<br>**3**: Don't know	**C8. Dustbin inside?**<br>**1**: Yes<br>**2**: No<br>**99**: Don't Know	**C11. Access for Disabled?**<br>**1**: Yes<br>**2**: No<br>**99**: Don't Know	**Notes**

C2. Are there separate toilets/latrines for teachers?

**Toilet units**	**C1. Toilet Type**<br>**1**: Flush/ pour flush<br>**2**: Pit<br>**3**: Bucket<br>**88**: Other: _____	**C2. User**<br>**1**: Women ONLY<br>**2**: Men ONLY<br>**3**: Both<br>**99**: Don't know	**C3. Functionality**<br>**1**: Functional<br>**2**: Partially<br>**3**: Not Functional<br>**99**: Don't Know	**C4. Cleanliness**<br>**1**: Clean<br>**2**: Somewhat<br>**3**: Not Clean<br>**99**: Don't Know	**C5. Lighting**<br>**1**: Light<br>**2**: Somewhat<br>**3**: Dark<br>**99**: Don't Know	**C6. Locks**<br>**1**: Yes, functional<br>**2**: Not functional<br>**3.** No<br>**99**: Don't Know	**C7. Access the toilet without assistance?**<br>**1**: Yes<br>**2**: No<br>**3**: Don't know	**C8. Dustbin inside?**<br>**1**: Yes<br>**2**: No<br>**99**: Don't Know	**C11. Access for Disabled?**<br>**1**: Yes<br>**2**: No<br>**99**: Don't Know	**Notes**

D. Waste disposal and drainage observations This section about disposal is pertinent to MHM because the ability to dispose of menstrual waste products easily and discretely is integral to a girls comfort when using a schools' toilet facilities.

D1. At the time of the visit, is there a facility destined for waste disposal? (e. g. waste pits/composting chambers/septic tanks)

D2. Is there a separate pit for burning used sanitary towels?

D3. Does the school have a drainage system for removing wastewater from the school grounds?

E. Hygiene observations This portion of the survey is about hand-washing facilities.

E1. Does the school have hand-washing facilities?

**Hand-washing Station #**	**E2. Hand-washing facility type**<br>1: Running water form<br>2: Hand-poured water system<br>3: Basin/bucket<br>88: Other (please specify)	**E3. Location of facility.**<br>1: Inside/ toilet<br>2: In classrooms<br>3: Close to cooking areas<br>5: Inside toilet stalls<br>88: Other	**E4. Water available *currently*?**<br>1: Yes<br>2: No	**E5. *Soap or ash* available *currently*?**<br>1: Yes<br>2: No	**E6. Is the hand-washing station accessible to students with disabilities?**<br>1: Yes<br>2: No	**Notes**

Additional notes on the visit:

[i] This survey has been adapted from the template provided by Emory University and UNICEF and both of these organizations will be, therefore, accredited in all future publications.

Appendix 4: School assessment additional data

Table 1: School overview

School Nr.	Location	School type	Schooling level	Nr. of students	Nr. of girls (%)	Nr. of teachers	Nr. of female teachers (%)
1	Semi-urban	Private	Secondary + High school	686	297 (43.29%)	42	7 (16.66%)
2	Semi-urban	Public	Secondary + High school	1,714	827 (48.24%)	61	17 (27.86%)
3	Semi-urban	Private	Secondary + High school	1,057	463 (43.80%)	30	8 (26.66%)
4	Semi-urban	Public	Secondary	540	304 (56.29%)	11	4 (36.36%)
5	Semi-urban	Private	Secondary + High school	351	128 (36.46%)	23	1 (4.34%)
6	Semi-urban	Private	Secondary*+ High school	281	123 (43.77%)	22	2 (9.09%
7	Rural	Public	Secondary	560		8	2 (25%)
8	Rural	Public	Secondary	311		4	0 (0%)
9	Rural	Public	Secondary	303		5	0 (0%)
10	Rural	Public	Secondary	244		5	0 (0%)
11	Rural	Public	Secondary	165	251 (44.82%)	5	2 (40%)
12	Rural	Public	Secondary	348	135 (43.40%)<br>150 (49.50%)<br>114 (46.72%)<br>55 (33.33%)<br>164 (47.12%)<br>63 (44.05%)<br>106 (56.08%)<br>3,180 (46.14%)	9	3 (33.33%)
13	Rural	Public	Secondary	143		6	1 (16.66%)
14	Semi-urban	Private	Secondary	189		9	3 (33.33%)
**Total**				6,892			
**Mean**					45.49%		20.41 %

*This school only offered the last year of secondary school

Table 2: Evaluation of students' toilets

School Nr.	Toilet type (Nr. of toilets in use)	Pupil:toilet ratio	Sexdivided	Girl:toilet ratio	Overall cleanliness (1-5)*	Lighting	Functional doors and locks
1	Pit (20)	34.3:1	Partial**	29.7:1	3	Dark	Yes
2	Pit (5)	342.8:1	No	N/A	3	Natural	Broken doors
3	Pit (3)	352.3:1	No	N/A	3	Natural	No door
4	Pit (3)	140:1	Yes	152:1	5	Dark	Yes
5	Pit (6)	58.5:1	No	N/A	5	Dark	Yes (1 no door)
6	Pit (6)	46.8:1	No	N/A	3	Natural	No door
7	Pit (6)	93.3:1	Yes	83.6:1	5	Dark	Yes (4 of 6)
8	Pit (1)	311:1	No	N/A	3	Natural	No door
9	Pit (4)	75.7:1	Yes	75:1	4	Natural	Yes
10	Pit (4)	62:1	Yes	57:1	5	Natural	Yes (1 no door)
11	Pit (4)	41.2:1	Yes	27.5:1	5	Natural	Yes
12	Pit (6)	58:1	No	N/A	5	Dark	Yes (4 of 6)
13	Pit (4)	35.7:1	No	N/A	5	Natural	Mostly not functional locks
14	Pit (2)	94.5:1	No	N/A	5	Natural	Yes

* Cleanliness was rated with the following scale: 1 – clean; 2 – somewhat clean, no smell; 3 – somewhat dirty and/or some smell; 4 – strong smell and/or dirty enough to discourage use; 5 – trash, excrements, urine and/or used toilet paper outside of the pit in at least one of the toilets.

**In this case, one toilet block was sex-divided and another one was not.

N/A – Not applicable.

Table 3: Evaluation of teachers' toilets

School Nr.	Toilet type (Nr. of toilets in use)	Sexdivided	Teacher:toilet (female teacher:toilet) ratio	Overall cleanliness (1-5)*	Lighting	Functional doors and locks

1	Flush (2)	Yes	21:1 (7:1)	1	Artificial	Yes
2	Flush (1)	No	61:1	3	Dark	Yes
3	Pit (2)	Yes	15:1 (8:1)	2	Dark	Yes
4	Pit (3)	No	3.6:1	2	Dark	Yes
5	Pit (1)	No	23:1	2	Natural	Yes
6	N/A	N/A	N/A	N/A	N/A	N/A
7	Pit (2)	No	8:1	3	Dark	Yes
8	N/A	N/A	N/A	N/A	N/A	N/A
9	Pit (1)	No	2.5:1	2	Natural	Yes
10	Pit (1)	No	5:1	3	Natural	Yes
11	Pit (2)	No	2.5:1	3	Natural	Yes
12	Pit (2)	Yes	4.5:1(3:1)	3	Dark	No
13	N/A	N/A	N/A	N/A	N/A	N/A
14	N/A	N/A	N/A	N/A	N/A	N/A

* Cleanliness was rated with the following scale: 1 – clean; 2 – somewhat clean, no smell; 3 – somewhat dirty and/or some smell; 4 – strong smell and/or dirty enough to discourage use; 5 – trash, excrements, urine and/or used toilet paper outside of the pit in at least one of the toilets. N/A – Not applicable, no toilets reserved for teachers.
